# A Novel Alpha Kinase EhAK1 Phosphorylates Actin and Regulates Phagocytosis in *Entamoeba histolytica*

**DOI:** 10.1371/journal.ppat.1004411

**Published:** 2014-10-09

**Authors:** M. Shahid Mansuri, Sudha Bhattacharya, Alok Bhattacharya

**Affiliations:** 1 School of Life Sciences, Jawaharlal Nehru University, New Delhi, India; 2 School of Environmental Sciences, Jawaharlal Nehru University, New Delhi, India; University of Virginia Health System, United States of America

## Abstract

Phagocytosis plays a key role in nutrient uptake and virulence of the protist parasite *Entamoeba histolytica*. Phagosomes have been characterized by proteomics, and their maturation in the cells has been studied. However, there is so far not much understanding about initiation of phagocytosis and formation of phagosomes at the molecular level. Our group has been studying initiation of phagocytosis and formation of phagosomes in *E. histolytica,* and have described some of the molecules that play key roles in the process. Here we show the involvement of EhAK1, an alpha kinase and a SH3 domain containing protein in the pathway that leads to formation of phagosomes using red blood cell as ligand particle. A number of approaches, such as proteomics, biochemical, confocal imaging using specific antibodies or GFP tagged molecules, expression down regulation by antisense RNA, over expression of wild type and mutant proteins, were used to understand the role of EhAK1 in phagocytosis. EhAK1 was found in the phagocytic cups during the progression of cups, until closure of phagosomes, but not in the phagosomes themselves. It is recruited to the phagosomes through interaction with the calcium binding protein EhCaBP1. A reduction in phagocytosis was observed when EhAK1 was down regulated by antisense RNA, or by over expression of the kinase dead mutant. G-actin was identified as one of the major substrates of EhAK1. Phosphorylated actin preferentially accumulated at the phagocytic cups and over expression of a phosphorylation defective actin led to defects in phagocytosis. In conclusion, we describe an important component of the pathway that is initiated on attachment of red blood cells to *E. histolytica* cells. The main function of EhAK1 is to couple signalling events initiated after accumulation of EhC2PK to actin dynamics.

## Author Summary

*Entamoeba histolytica* is one of the major causes of morbidity and mortality in developing countries. Phagocytosis plays an important role in both survival and virulence, and has been used as one of the virulence markers. *E. histolytica* displays a high rate of phagocytosis and offers a unique system to understand the mechanism of this important biological process seen in many eukaryotic cells. However, the molecular mechanism of the process is still largely unknown in *E. histolytica,* though this pathway has been characterized in many systems. We have been studying this pathway using red blood cells, and have identified a number of molecules that are involved during initiation. Here, we demonstrate that an alpha kinase like atypical kinase EhAK1 is an important component of the pathway that regulates erythrophagocytosis. We provide evidence that EhAK1 is recruited to the phagocytic cups through EhCaBP1. We also show that over expression of kinase defective mutant, or down regulation of the gene using antisense RNA, led to defects in phagocytosis. Actin appears to be one of the substrates of EhAK1 and phosphorylation of actin is required for phagocytosis. Our results suggest that *E. histolytica* has evolved a novel pathway to carry out phagocytosis.

## Introduction

Phagocytosis is an essential process both in unicellular organisms which use this process to obtain their food [1], and multicellular organisms, where it plays a central role in the innate immune system [2]. In the protist pathogen *Entamoeba histolytica* it is an important process for nutrient uptake as well as for amoebic invasion. Cells lose their ability to invade when phagocytosis is inhibited [3]–[4]. However, the mechanism of phagocytosis, particularly the initial steps leading to phagocytic cup formation up to phagosome closure in *E. histolytica*, is not clearly understood unlike metazoan systems where the process has been studied in extensive detail [5]–[6]. Phagocytosis in *E. histolytica* is likely to follow a different molecular path compared to mammals as a number of molecules known to be involved in mammalian phagocytosis could not be identified in this organism [7]. A number of cell surface molecules, such as Gal/GalNAc lectin [8], TMK96 [9], TMK39 [10], SREHP [11] and EhROM1 [12] have been shown to be involved in adherence to other cells. It is not yet clear if these molecules are amoebic receptors during phagocytosis of different particles, such as RBC, bacteria and apoptotic human cells [13]. The participation of Gal/GalNAc lectin as a receptor in phagocytosis has been questioned, though it is likely that it may still be a key molecule initiating signal transduction [14]–[15]. Analysis of the phagosome proteome has revealed involvement of a large number of proteins in phagosome formation and subsequent maturation [14], [16]–[19]. Some of these, such as actin [20], Arp proteins, actin binding proteins, PI3 kinase [21], P21 activated protein kinase (PAK) [22], and different GTPases are already known to be part of phagocytic and signalling pathways [23]–[24]. A transmembrane kinase PATMK was identified from one of the proteomic screens of phagosomes [9]. Detailed analysis suggested that PATMK is localized at the site of RBC attachment to *E. histolytica* cells and that it is involved in phagocytosis. Though many of the identified molecules are suggested to be part of the phagocytic pathways, detailed molecular mechanisms have not yet been elucidated. Myosin 1B has also been suggested to be one of the key molecules in phagocytosis of human cells [25]. Over expression of myosin 1B reduces phagocytic capabilities of amoebic cells, probably through altering the level of actin network [26]. Previous studies from our laboratory have shown that calcium binding protein1 (EhCaBP1) is involved in the initiation of phagocytosis [27]. EhCaBP1 is recruited to the phagocytic cups with the help of a C2 domain-containing protein kinase (EhC2PK). EhC2PK binds phosphatidylserine-containing membranes in the presence of Ca^2+^ through its C2 domain. Both EhCaBP1 and EhC2PK are likely to be involved in cup progression through recruitment of proteins that regulate actin dynamics, and both of these proteins leave phagocytic cups before closure [28]. A calmodulin-like calcium binding protein EhCaBP3 has also been implicated to participate in erythrophagocytosis [29]. It is recruited during cup formation and stays till phagosomes are formed. EhCaBP3 binds atypical myosin 1B, and is suggested to be involved in phagosome closure.

The spatial and temporal regulation of actin dynamics is the key for controlling phagocytosis. Blocking actin dynamics by inhibitors leads to a reduction in phagocytosis [30]. Though this area has been extensively investigated in metazoans, not much is known in protist parasites, particularly *E. histolytica*. Only the homologs of a few molecules known to play important roles in other systems have been identified, mostly using bioinformatics tools [31]. The participation of Ca^2+^ in phagosomal closure and maturation has been suggested and it is believed that interaction of a particle with phagocytic receptors generates Ca^2+^ oscillations in the cytoplasm, which cause solubilisation and periphagosomal breakdown of actin filaments surrounding the phagosomes [32]. Ca^2+^ signal may also regulate actin dynamics through Ca^2+^ binding proteins (CaBPs) that can sense alteration in Ca^2+^ concentration and modulate actin filaments [33]. In *Dictyostelium discoideum*, two CaBPs (34 kD and 40kD) are involved in bundling and cross-linking of actin filaments [34]–[35]. Overall, the localized control of actin dynamics around the phagocytic cups helps to generate a force that propels psuedopod movement around the particles, leading to engulfment. The mechanisms that regulate actin dynamics in *E. histolytica* are likely to be somewhat different as amoebic actin displays some unusual properties. Amoebic actin can polymerize to form filaments *in vitro* and the polymerized actin can induce myosin ATPase displaying kinetics similar to that of rabbit actin [36]. However unlike other actin, this actin did not bind DNase I or polymerize at low temperature. Moreover, amino acid sequence of amoebic actin showed 10–15% differences compared with different mammalian actins, suggesting that functional differences may be due to sequence variations. However, antibodies to the purified amoebic actin recognized actin from several eukaryotes including protozoa indicating that there may be overall conformation conservation among different actins [3], [5]–[7], [31], [36].

It is clear from previous studies that the mechanisms regulating phagocytosis in different systems may depend upon the nature of particle or ligand. Therefore we have used only RBC uptake as a system to study molecular mechanism of initiation and propagation of the phagocytic signal. Here we describe the functional characterization of a novel alpha kinase-like kinase EhAK1. Alpha-kinases are a class of atypical protein kinases, characterized by unusual substrate specificity, and the absence of significant sequence similarity to conventional protein kinases [37]. These kinases phosphorylate serine and threonine residues in the context of an alpha-helix present in the substrate. Many alpha kinases carry domain(s) other than alpha kinase domain, allowing participation in a variety of processes, such as protein translation, Mg^+2^ homeostasis, intracellular transport, cell migration, and proliferation. Some of the domains associated with alpha kinases are channel, Ig, CaM, WD40 repeat and Arf-Gap (For a review see Middlebeek et al, 2010). In this report, we show that EhAK1 is required for the initiation of phagocytosis in *E. histolytica*. Our results indicate that erythrophagocytosis is initiated by the recruitment of EhAK1 with the help of EhCaBP1 at the RBC attachment site, which is followed by recruitment of actin. We also show that actin is a substrate of EhAK1 unlike other alpha kinases which phosphorylate myosin [38]. Our data suggest that actin phosphorylation is likely to be an important mechanism for regulating localized actin dynamics.

## Results

### EhAK1 is a cytosolic protein and is involved in erythrophagocytosis

**Figure 1:**
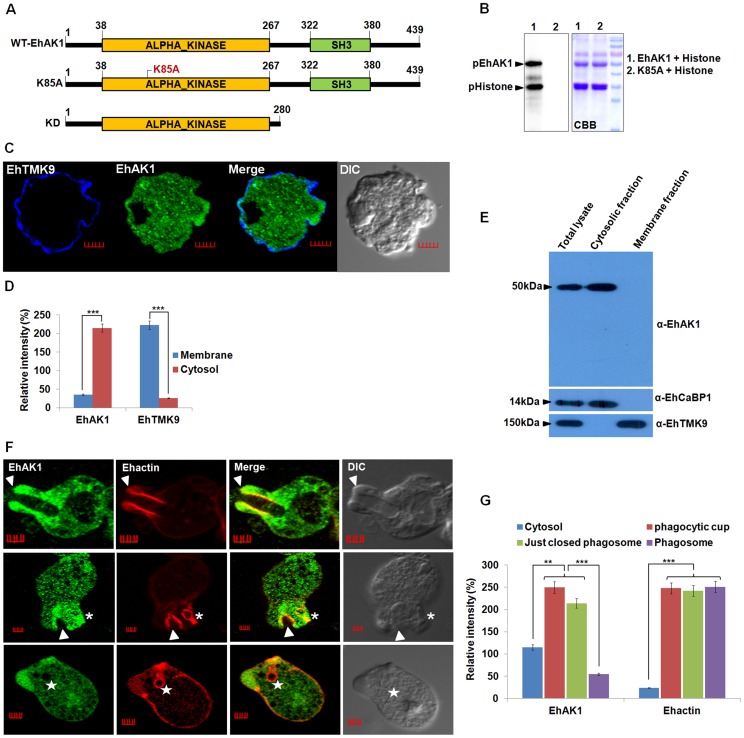
EhAK1 is involved in phagocytosis. (A) Schematic representation of domain organization of EhAK1. EhAK1 is a 50 kDa protein with two domains, alpha kinase domain (38–267 amino acids) and SH3 domain (322–380 amino acids). The three different constructs used are shown. Lys 85 is the nucleoside triphosphate binding site, K85A mutant is the kinase dead mutant, KD-kinase domain alone. (B) Purified recombinant EhAK1 or K85A (2µg) was incubated in the presence of γ-32P-ATP, MgCl_2_ and substrate (2µg) histone type (IIIS) at 30°C for 1 h. K85A mutant of EhAK1 exhibits no autophosphorylation and substrate phosphorylation activities. The products were analysed on SDS-PAGE and visualized in a phosphorimager. (C) Amoebic cells were stained for EhAK1 and EhTMKB1-9 (as membrane marker) using specific antibodies followed by Alexa488 and pacific blue-410, respectively. (D) Quantitative analysis of fluorescent signals obtained from panel (C). Intensity of immunostain (EhAK1, EhTMKB1-9) was measured at multiple locations in the membrane and cytosol and average relative intensity was computed by taking the signal from membrane as 100% for each marker separately. For analysis, five random regions were selected from membrane (blue) and cytosol (red) and average intensity was computed for each region. This was repeated for ten such cells (N = 10, bars represent standard error; scale bar, 5 µm). (E) Subcellular localization of EhAK1. *E. histolytica* whole cell lysate prepared from mid log phase cells, was fractionated biochemically in to cytoplasmic and membrane fractions as described in “Material and Methods”. Fifty micrograms of protein of indicated fraction was separated on SDS-PAGE, electrophoretically transferred to PVDF membranes and then immunostained with anti-EhAK1, anti-EhCaBP1 (cytoplasmic) and anti-EhTMK9 (membrane) antibodies as shown. (F) Imaging of EhAK1 and actin during erythrophagocytosis. *E. histolytica* cells were grown for 48 h and incubated with RBC for different times at 37°C. Immunostaining was performed using anti-EhAK1 antibody followed by Alexa-488. F-actin was stained with TRITC-phalloidin. Arrowheads indicate phagocytic cups, asterisks mark just closed cups (before cessation) and star marks phagosome. Bar represents 5 µm. (G) Quantitative analysis of fluorescent signals obtained by immunostaining of EhAK1 from different locations in *E. histolytica* cells (N = 5) as described in panel (D). *p-value≤0.05, **p-value≤0.005, ***p-value≤0.0005.

Our previous work has shown the importance of EhCaBP1 in RBC phagocytosis. Signalling through EhCaBP1 after attachment of RBCs appears to be crucial for progression of phagocytic cups towards phagosomes [27], [39]. In order to identify the molecules that may be involved in phagocytosis along with EhCaBP1 we had carried out affinity chromatography of amoebic extract on an EhCaBP1-Sepharose column [28]. Mass spectrometry of bound molecules helped to identify a number of proteins, such as actin, myosin and EhC2PK. The functional role of EhC2PK during initiation of phagocytosis has already been described [28]. A homolog of alpha kinase EhAK1 was also identified as one of the EhCaBP1 binding proteins. Myosins play a critical role in manipulating actin cytoskeleton function. Since alpha kinases are known to control myosin through phosphorylation [40]–[41], we decided to pursue EhAK1 as a potential regulator of phagocytosis-related actin dynamics. *E. histolytica* encodes five alpha kinases, of which EhAK1 and EhAK2 have unusual domain organization, with alpha kinase domains at the N-terminus, and SH3 domains at the C-terminus **(Fig. S1)**. Association of a SH3 domain with alpha kinase catalytic domain has not been seen among the known alpha kinases [42]. The kinase domain of the other three alpha kinases was located at C-terminal end and there was no other recognisable domain in these molecules. EhAK1 and EhAK2 displayed low level of overall sequence conservation with other alpha kinases **(Fig. S1)**. Sequence similarity search using the kinase domain of EhAK1 showed maximum identity (30%) with alpha kinases from *D. discoideum*. The kinase domain has eight conserved sub domains and a zinc-finger motif at its C-terminus, which is present in all known alpha kinases [42] **(Fig. S2)**. Multiple alignments of different alpha kinases helped to identify conserved residues in the EhAK1 kinase domain, including lysine at 85^th^ position that plays an important role in binding nucleoside triphosphate. Mutation of this residue to alanine abolished the catalytic activity of EhAK1 **(** **Fig. 1A and 1B** **)**.

The sub-cellular distribution of EhAK1 was investigated by using a specific antibody raised against the recombinant protein **(Fig. S3 and S4A)**. As a control we used antibodies against a known membrane marker EhTMKB1-9 [43]. It is clear that the majority of EhAK1 is present in the cytosol, unlike EhTMKB1-9 and that there is very little colocalization in the membrane **(** **Fig. 1C-D** **)**. This was further confirmed by cell fractionation followed by identification of proteins by immunoblotting (**Fig. 1E**). Distribution of EhAK1 was similar to that of EhCaBP1, a cytoplasmic protein [44]. It was found in the cytoplasmic fraction but not in the membrane fraction. On the other hand the cell surface protein EhTMKB1-9 was detected only in the membrane fraction. Localization of GFP-tagged full length EhAK1 or the kinase domain (KD) alone, displayed a pattern similar to that seen for untagged EhAK1 **(Fig. S4B-C)**. We have also analysed cells expressing GFP-EhAK1 using antibodies against both GFP and EhAK1 and quantified signals in 5 randomly chosen spots within cytoplasm and the results showed colocalization of signals from both stains **(Fig. S4D)**. The results suggest that the GFP tag does not affect the properties of EhAK1. In order to study the distribution of EhAK1 during erythrophagocytosis *E. histolytica* cells were incubated with human RBCs for different times, and then processed for visualization after staining. Actin was also stained with phalloidin in order to identify cups and phagosomes. **Fig 1F** shows images of representative cells at different stages of phagocytosis. EhAK1 was found in the phagocytic cups (marked with an arrow), and in just closed phagosomes, that is, phagosomes after sealing but before cessation (marked with an asterisk). It was not found in phagosomes after cessation (marked with a star). Quantification of EhAK1 at different stages of phagocytosis was done by measuring signals from 10 different cells. The results clearly showed that EhAK1 is enriched in phagocytic cups and it stays there till the phagosomes separate out from plasma membrane **(** **Fig. 1G** **)**. F-actin and EhAK1 appeared to be colocalized at the phagocytic cups and phagosomes before cessation, as confirmed using Pearson correlation coefficient (r = 0.82±0.2).

**Figure 2:**
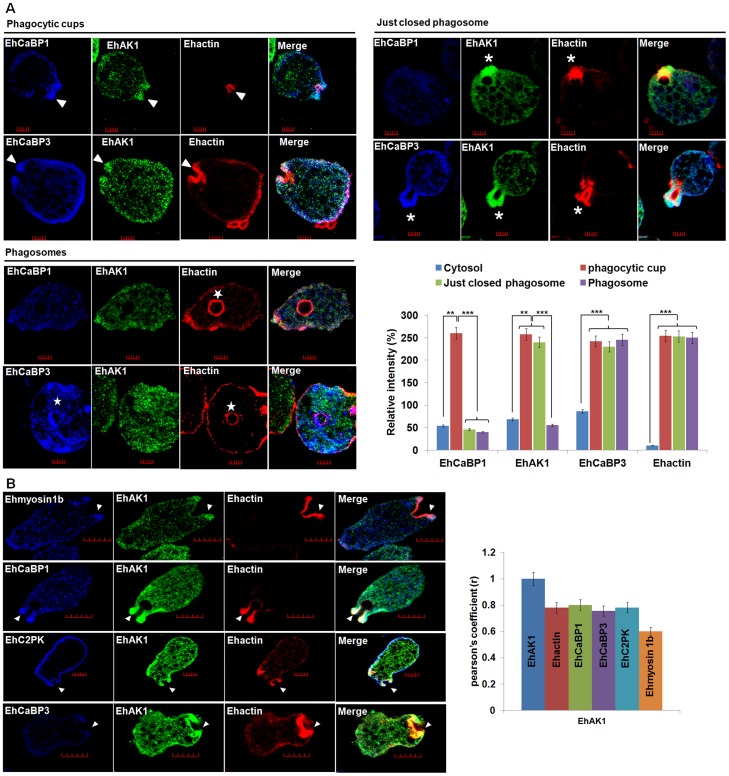
Colocalization of EhAK1 with EhMyosin 1B, EhC2PK, EhCaBP1 and EhCaBP3 at the phagocytic cup during erythrophagocytosis. (A) Imaging of EhAK1, EhCaBP1 and EhCaBP3 during erythrophagocytosis. *E. histolytica* cells were incubated with RBC for 5 min at 37°C. The cells were then fixed and immunostained with anti-EhAK1 antibody followed by Alexa-488. F-actin was stained with TRITC-phalloidin and other indicated proteins were immunostained with respective antibodies and followed by Pacific blue-410. Arrowheads indicate phagocytic cups, asterisks mark just closed cups and star marks phagosome. Bar represents 5 µm. Quantitative analysis of fluorescent signals obtained by immunostaining of EhAK1, EhCaBP1 and EhCaBP3 from different locations in *E. histolytica* cells (N = 5) was done as described in Fig. 1D. (B) The incubation and labelling conditions were as described in Fig. 1. Ehmyosin 1B, EhC2PK, EhCaBP1 and EhCaBP3 were immunostained with specific antibodies and visualized using Pacific blue-410 (blue). Colocalization analysis from five cells was done by using JACoP (ImageJ). The Pearson's coefficient (r) of EhAK1 with EhAK1, EhCaBP1, EhCaBP3, Ehmyosin 1B and EhC2PK from phagocytic cups are indicated. *p-value≤0.05, **p-value≤0.005, ***p-value≤0.0005.

In order to investigate the participation of EhAK1 in the phagocytic process in relation to the three known proteins,(EhCaBP1, EhCaBP3 and EhC2PK) we carried out immunostaining experiments in a pair-wise manner and analyzed the images both visually as well as quantitatively, based on intensity of stain in different compartments **(** **Fig. 2A** **)**. The distribution of EhAK1 was found to be different from that of EhCaBP1 and EhCaBP3. While EhCaBP1 left phagocytic cups before closure of the cups, EhCaBP3 remained till the phagosomes separated from the plasma membrane **(** **Fig. 2A** **)**. Although EhAK1 colocalized with EhCaBP1, EhCaBP3, EhC2PK, Ehmyosin1B and actin at the cups **(** **Fig. 2B** **)**, its distribution was distinctly different with respect to the other molecules in just-closed phagosomes and phagosomes after cessation. Therefore it appears that the role of EhAK1 in phagocytosis may be different from the other indicated molecules.

**Figure 3:**
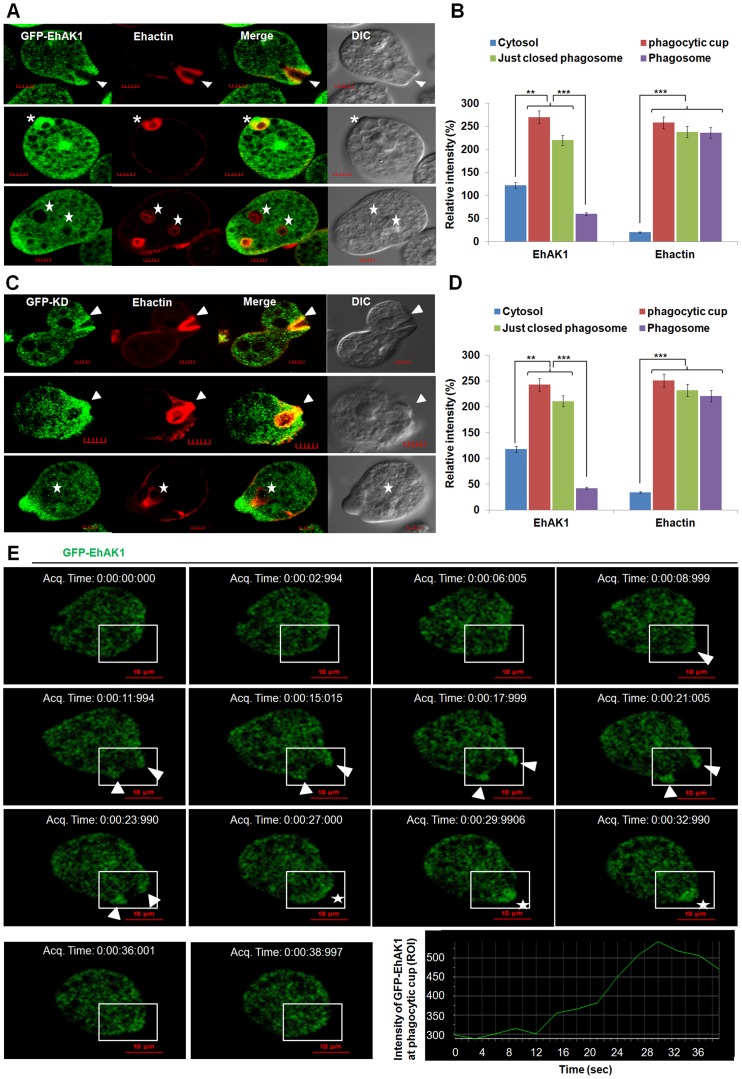
*In vivo* localization of GFP-EhAK1 in phagocytosing cells. (A) and (C) Imaging of GFP-EhAK1 and GFP-KD and colocalization with actin during erythrophagocytosis. GFP-EhAK1 and GFP-KD expressing cells were grown for 48h with 30µg/ml G418 and incubated with RBC for different time at 37°C. Immunostaining was performed using anti-GFP antibody followed by Alexa-488. F-actin was stained with TRITC-phalloidin. EhCaBP1 was immunostained with specific antibodies and visualized using Pacific blue-410 (blue). Arrowheads indicate phagocytic cups, asterisks mark just closed cups and star marks phagosome. Bar represents 5 µm. (B) and (D) Quantitative analysis of fluorescent signals was done as in Fig. 1D. (E) Time lapse imaging of GFP-EhAK1. The montage shows a time series of GFP-EhAK1 expressing cells undergoing erythrophagocytosis where phagocytic cups are marked by arrowhead and just closed phagosomes by star. Bar represents 10 µm. Graph shows the intensity of GFP-EhAK1 (ROI) at a phagocytic cup every 3s. *p-value≤0.05, **p-value≤0.005, ***p-value≤0.0005.

To enable visualization by live cell imaging, we used cell-lines expressing GFP-tagged EhAK1. As mentioned earlier, the distribution of GFP-EhAK1 was similar to endogenous EhAK1. GFP-EhAK1 was also colocalized with actin and was absent in phagosomes after cessation, but present in phagosomes before cessation **(** **Fig. 3A–B** **)**. Moreover, we have analysed cells expressing GFP-EhAK1 or GFP using antibodies against both GFP and EhAK1. Results showed colocalization of signals from both stains at phagocytic cups in cells over-expressing GFP-EhAK1. We did not observe any GFP signal at phagocytic cup in cells over-expressing only GFP **(Fig. S4E)**. We also observed similar distribution for the GFP-tagged kinase domain (GFP-KD), reinforcing our earlier finding that GFP tag does not alter basic property of EhAK1 **(** **Fig. 3C–D** **)**. Live cell imaging of GFP-EhAK1 expressing cells undergoing erythrophagocytosis showed that EhAK1 accumulated rapidly at the site of RBC attachment and remained there till closure of the cups. EhAK1 was absent in phagosomes **(** **Fig. 3E** **and Movie S1)**. Quantitative analysis of the fluorescence signal (ROI) showed significant accumulation by about 17s, reaching maximum intensity during closure of the cup at about 30s. Subsequently signal intensity underwent a gradual decline. It took about 38 s from the first appearance of EhAK1 to its eventual disappearance from the cups **(** **Fig. 3E** **)**. In contrast there was no significant change in EhAK1 fluorescence intensity in the cytoplasm during this period.

**Figure 4:**
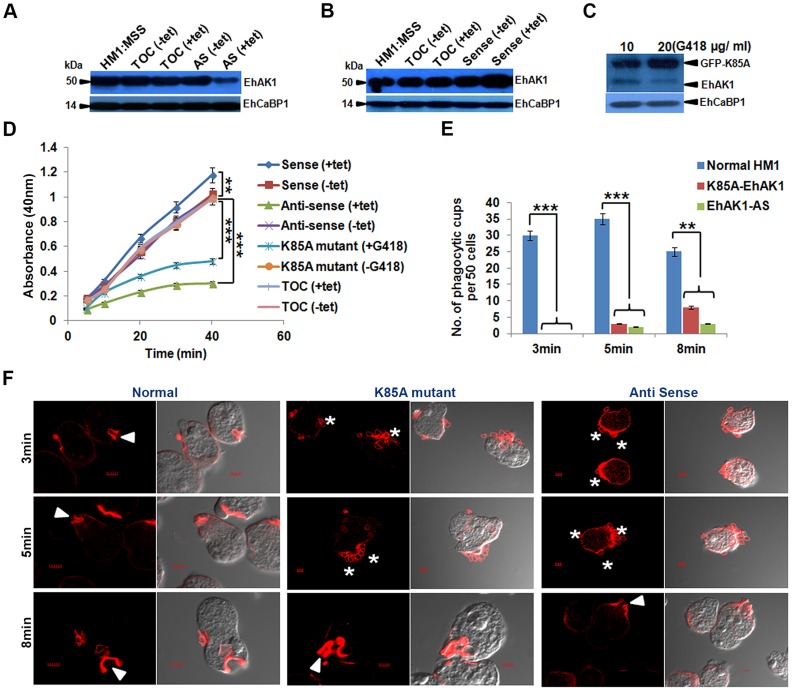
EhAK1 is involved in the initiation of phagocytosis. (A) and (B) Fifty microgram of total cell lysate from indicated *E. histolytica* cell-lines was analyzed by western blotting to measure the levels of EhAK1. EhCaBP1 was used as an internal control. Cells (HM1:IMSS) were transfected with tet-inducible vector alone, antisense EhAK1 (AS) or sense EhAK1 (sense). Tetracycline (30 µg/ml) was added to induce gene expression in the +tet lanes. TOC is tet-o-CAT vector and AS is antisense. (C) Western blot analysis (as in panels, A and B) with cells carrying GFP-K85A construct of EhAK1 cloned in a constitutive vector. Cells were grown at the indicated concentration of G418. (D) RBC uptake assay was performed using the indicated cells grown with or without tet or G418. The experiments were carried out three times independently in triplicates. ANOVA test was used for statistical comparisons. (E) Quantitative determination of phagocytic cups observed in the indicated cell-lines was carried out by randomly selecting fifty cells in each experiment, and counting the number of phagocytic cups present in all these cells. (F) Cells were incubated with RBCs for the indicated time at 37°C. Cells were then fixed and stained for actin with TRITC-Phalloidin, or immunostained with anti-EhAK1 antibody followed by Alexa-488. The accumulation of actin at phagocytic cups is marked by solid arrowheads. Asterisks show attached RBCs at the site of phagocytosis. *p-value≤0.05, **p-value≤0.005, ***p-value≤0.0005

Next we generated conditional (tet-inducible) EhAK1-knockdown cells by inducible expression of EhAK1 antisense RNA [27]. Western blot analysis showed that over-expression of antisense EhAK1 RNA in the presence of tet reduced the level of EhAK1 by 70 to 75% in multiple replicates, as determined by densitometric scanning **(** **Fig. 4A** **)**. EhCaBP1 levels were also determined in these cells to ensure that any changes in phagocytic properties of EhAK1-AS cells may not be attributable to altered leads of EhCaBP1 which has a known role in phagocytosis [27]. There was no significant change in the level of EhCaBP1 in these cells suggesting that the down regulation of EhAK1 was specific **(** **Fig. 4A** **)**. The down regulation of EhAK1 was also visualized by immunostaining. Tet-induced antisense cells displayed a significant reduction (about 80%) in fluorescent signal as compared to vector control cells under similar conditions **(Fig. S5)**. Further we over-expressed EhAK1 and the kinase dead mutant (K85A-EhAK1) by cloning the genes in the sense orientation in the same vector system and GFP-vector respectively. Over-expression increased the level of EhAK1 protein by about 40 to 50% in multiple replicates **(** **Fig. 4B** **)**. Mutant protein was identified by GFP-tag and endogenous protein, by EhAK1 antibody in western blots. The ratio between these two proteins was estimated to be about 1.5 to 2.5 in different experiments **(** **Fig. 4C** **)**. Erythrophagocytosis was measured in these cell lines in the presence and the absence of tet using a colorimetric assay that quantifies total amount of phagocytosed RBC and using equal number of cells for all cases. It was reduced by 45 and 40% after 5 min, and 88 and 75% after 40 min of incubation with RBC in cells expressing antisense EhAK1 and K85A mutant gene respectively, compared with cells with only vector **(** **Fig. 4D** **)**. On the other hand phagocytosis in wild type EhAK1 over-expressing cells increased by 20% in presence of tet as compared to that in absence of tet **(** **Fig. 4D** **)**. The observed reduction in erythrophagocytosis may be due to an effect at any of the steps in the process, namely initiation, progression of cups or cessation of phagosomes. This was investigated by determining the number of cups or phagosomes formed at different time points. In wild type cells the number of cups almost reached saturation by 3 min (average 0.6 cups/cell) and did not increase after 8 min (average 0.5 cups/cell). On the other hand, very few cups were visible in mutant (average 0.06 cups/cell) and antisense cells (average 0.04 cups/cell) by 5 min. Interestingly, the number of cups increased over time in mutant cells but not in antisense cells, and by 8 min the cells showed a significant number of cups (average 0.16/cell respectively) **(** **Fig. 4E–F** **)**. Phagocytosis was also visualised by using fluorescent-labelled RBCs. Many phagocytosed RBCs were visible as green particles (arrow) inside amoebic cells. F-actin was also labelled with phalloidin (red). The two stains did not merge as the stains were in different compartments of phagocytosed RBCs; F-actin being outside and RBCs inside the phagosomes. We have analysed different optical sections of the images and the results show absence of merging of the two stains. Absence of F-actin label in many phagosomes is due to the loss of F-actin during the process of maturation **(Fig. S6A and B)**. Using this system we further observed that the number of phagocytosed RBCs decreased significantly by 70% and 65% in EhAK1-AS or mutant over-expressing cell lines as compare to control at 8 min **(Fig. S6C)**. Live cell imaging of phagocytosing fluorescent-RBC by cells over-expressing mutant EhAK1 showed attachment of RBCs, but a defect in uptake was noticed unlike normal cells **(Movie S2 and Movie S3)**. These results suggest that functional EhAK1 may be required for erythrophagocytosis. Similar results were obtained when cell proliferation was measured. A significant reduction in proliferation was observed in both antisense and mutant over-expressing cell lines **(Fig. S7)**, consistent with our previous finding with EhCaBP1 [27].

### EhAK1 is recruited to the phagocytic cups through EhCaBP1

**Figure 5:**
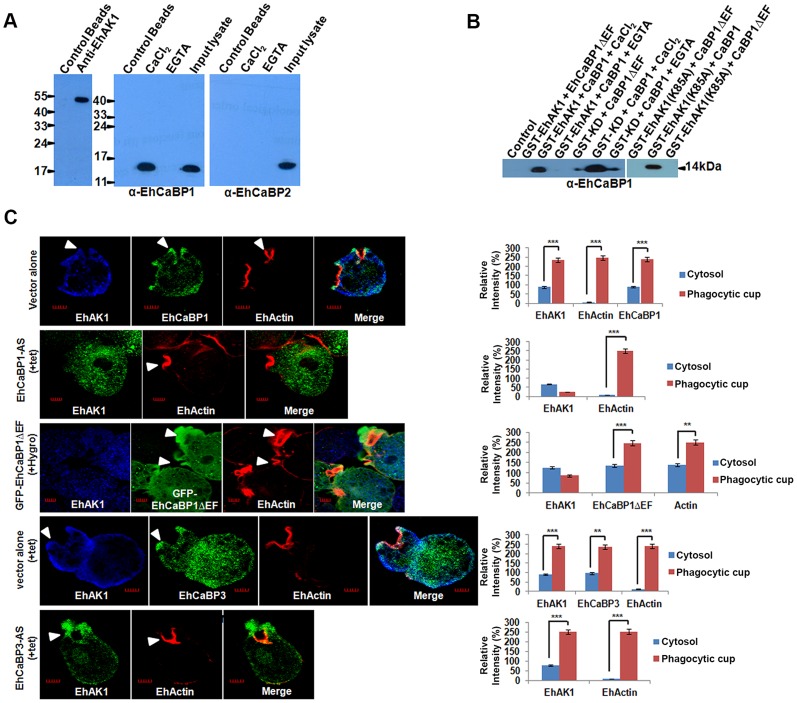
Recruitment of EhAK1 at the phagocytic cups through EhCaBP1. (A) Whole-cell lysate of *E. histolytica* was passed through agarose conjugated with either anti-EhAK1 antibody or preimmune serum. Co-IP of EhCaBP1 and EhCaBP2 was checked using respective antibodies. (B) Co-precipitation of EhCaBP1 or its calcium-binding defective mutant EhCaBP1ΔEF with GST-tagged protein (GST-EhAK1, GST-KD or GST-K85A) was carried out using Glutathione Sepharose beads. Proteins were detected using the anti-EhCaBP1 antibodies in western blots. (C) EhAK1 is recruited to the phagocytic cups through EhCaBP1. Cells containing indicated constructs were grown for 48 h in the presence of 30µg/ml tet or G418 and incubated with RBC for 5 min at 37°C. The cells were then fixed and immunostained with specific antibodies as indicated. F-actin was stained with TRITC-phalloidin. Arrowheads indicate phagocytic cups. Bar represents 5 µm. A few representative cells are shown. Graph shows quantitative analysis of fluorescent signals of immunostained images for (N = 5) cells as described in Fig. 1D. *p-value≤0.05, **p-value≤0.005, ***p-value≤0.0005.

The role of EhCaBP1 in recruitment of EhAK1 was investigated by immunoprecipitation and pull down experiments using either specific antibodies or affinity matrix for the GST-tag attached to the recombinant proteins. The EhAK1 antibody precipitated EhAK1 along with EhCaBP1 from the total cell lysate in the presence of Ca^2+^, but not in presence of EGTA **(** **Fig. 5A** **)**. This binding was specific as EhCaBP2, a protein with 79% identity at the amino-acid level with EhCaBP1, but with different function [45], was not detected in the immunoprecipitate **(** **Fig. 5A** **)**. This interaction was further confirmed *in vitro* using bacterially expressed EhAK1 as a GST-tagged protein. Binding of EhCaBP1 to glutathione-Sepharose beads was also observed with bacterially expressed K85A-EhAK1 mutant and with the kinase domain (KD) alone **(** **Fig. 5B** **)**. No binding was observed with a Ca^2+^-binding defective mutant of EhCaBP1 (CaBP1ΔEF) [39] **(** **Fig. 5B** **)**. Binding to EhCaBP1 was abolished in the presence of EGTA, demonstrating that Ca^2+^ is essential for interaction. Interestingly we observed a low level of binding of only the KD with EhCaBP1ΔEF or EhCaBP1 in the presence of EGTA, whereas no binding was observed when full length protein was used. This may be either due to of the conformation of the full length protein or a negative influence of SH3 domain during the interaction with EhCaBP1. We further confirmed the ability of the kinase defective mutant K85A-EhAK1 to bind EhCaBP1 by an *in vitro* competition assay. WT-EhAK1 was allowed to bind EhCaBP1 in the presence of increasing concentration of GST-K85A mutant and the complex was pulled down using anti EhCaBP1 antibody (see “Materials and Methods”). Results showed decreased binding with increasing concentration of K85A, suggesting that the mutant protein was capable of binding EhCaBP1 and competing with wild type EhAK1 **(Fig. S8)**. These experiments indicate that the kinase domain of EhAK1 binds EhCaBP1 in the presence of Ca^2+^. However, functional kinase domain is not necessary for this binding.

If EhAK1 is recruited through EhCaBP1 it is expected that EhAK1 recruitment will be reduced when EhCaBP1 levels go down. This was checked by down-regulating EhCaBP1 through antisense expression as described before [27]. Protein concentration in the cytosol and phagocytic cups was determined by immunostaining. Representative images are shown, and quantitative results are based on 5 different cells **(** **Fig. 5C** **)**. In cells transfected with vector alone, actin was seen mainly in phagocytic cups, while EhAK1 and EhCaBP1 were visible in both cytosol and phagocytic cups **(** **Fig. 5C** **)**. The ratio of signal in cups versus cytosol was 2.5 and 2.7 for EhAK1 and EhCaBP1 respectively. In EhCaBP1-antisense cells the overall signal was reduced and the ratio was negative, suggesting defective recruitment of EhAK1 to the phagocytic cups in the absence of EhCaBP1 **(** **Fig. 5C** **)**. In normal cells phagocytic cups are visible within a minute after the addition of RBCs. However, in antisense cells phagocytic cups began to appear only after 5 min and, compared with vector control, only 10% of the cells showed cup formation, suggesting that these cells are defective in cup formation **(Fig. S9)**. In cells over expressing GFP-EhCaBP1ΔEF [39], there was no enrichment of EhAK1, although the mutant EhCaBP1 was recruited to phagocytic cups **(** **Fig. 5C** **)**. The effect was specific to EhCaBP1, as in EhCaBP3 down-regulated cells there was no significant difference in the recruitment of EhAK1 (ratio of cups to cytosol, 2.5) **(** **Fig. 5C** **)**. These results suggest that EhAK1 is involved in phagocytosis and is recruited to phagocytic cups through EhCaBP1, in a Ca^2+^-dependent manner.

**Figure 6:**
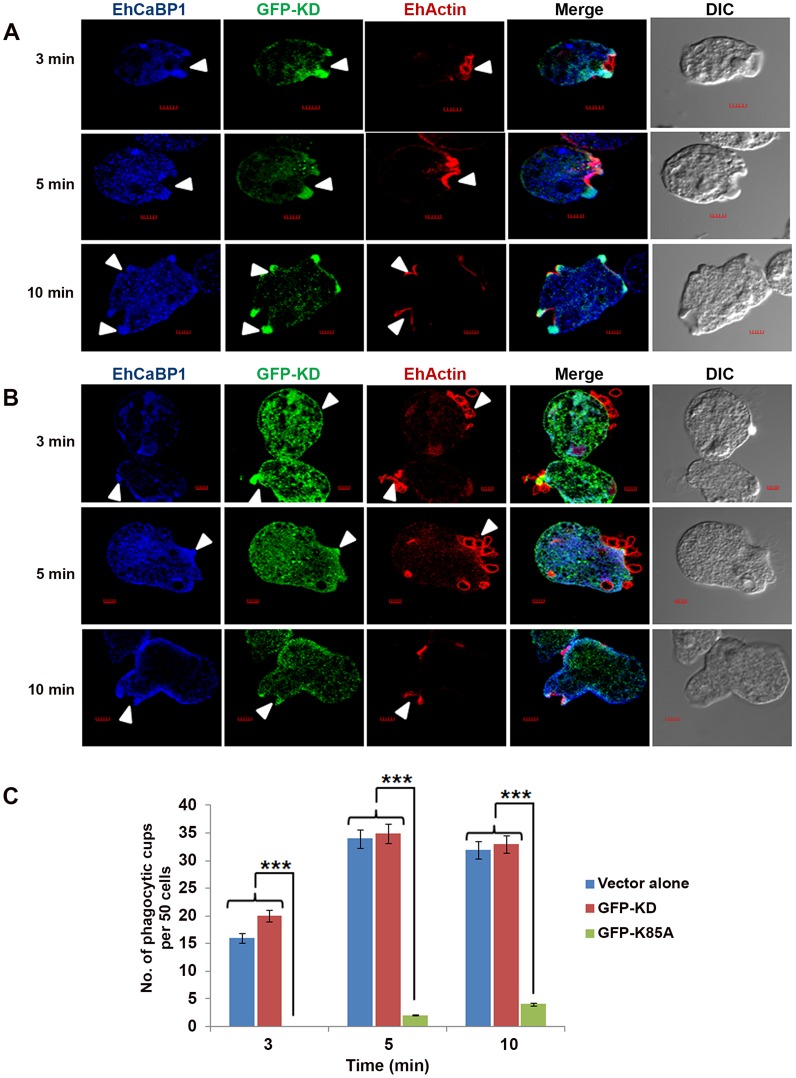
Functional kinase domain is essential for initiation of phagocytosis. (A) *and* (B) *E. histolytica* cells containing GFP-KD or GFP-K85A constructs were grown for 48h and incubated for the indicated times with RBCs. GFP-tagged proteins and EhCaBP1 were immunostained with anti-GFP and anti-EhCaBP1 antibodies respectively. Solid arrow shows phagocytic cups and enrichment of indicated proteins. Scale bar, 5 µm. (C) Quantitative analysis was carried out by selecting randomly fifty cells from each experiment and the numbers of phagocytic cups present in all cells were counted. *p-value≤0.05, **p-value≤0.005, ***p-value≤0.0005.

In order to see if functional kinase domain is required and sufficient for recruitment through EhCaBP1, cells expressing GFP-KD and GFP-K85A-EhAK1 were observed at different times during erythrophagocytosis. GFP-KD was seen in the cups along with EhCaBP1 and actin **(** **Fig. 6A** **)**. Interestingly, colocalization was not visible all around the cup; it was more prominent in the tips of expanding psuedopods **(** **Fig. 6A** **)**. In GFP-K85A-EhAK1 over expressing cells, phagocytic cup formation was not seen at 3 min though many RBCs were seen attached to amoebic cells. A few cups were visible at latter time points which displayed enrichment along with EhCaBP1 **(** **Fig. 6B** **)**. Examination of fifty such cells showed that the numbers of cups were reduced by 88% at 10 min **(** **Fig. 6C** **)**. These results indicate that KD alone can interact with EhCaBP1 and initiate cup formation, and that kinase activity is required for stabilization and progression from cups to phagosomes.

### EhAK1 phosphorylates G-actin at Thr 107

**Figure 7:**
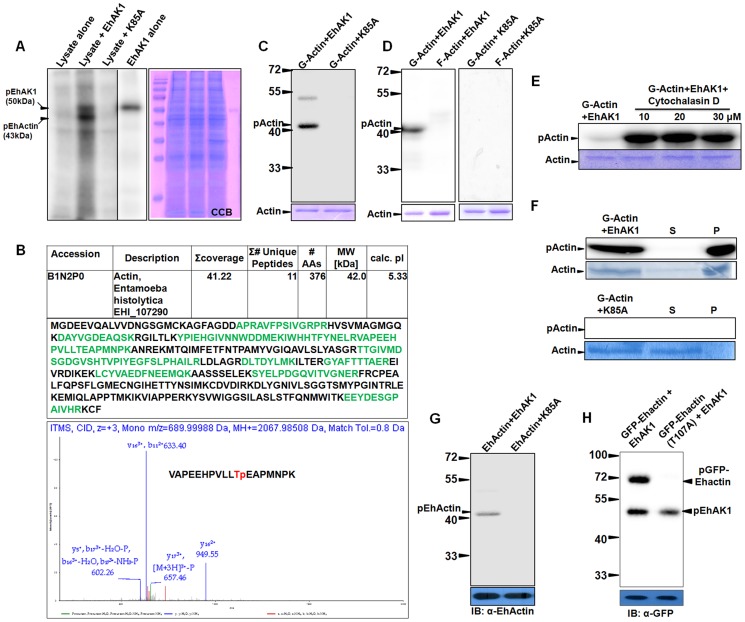
EhAK1 phosphorylates G-actin at threonine 107. All phosphorylation reactions were carried out *in vitro* and products were resolved on SDS-PAGE. Radiolabeled products were visualized in a phosphorimager. (A) Phosphorylation of Ehactin by EhAK1. Total cell lysate (200µg) was incubated with γ-^32^P-ATP in the presence of EhAK1 (2µg). Reaction was stopped with SDS sample buffer containing EDTA after boiling and samples were resolved on 12% SDS-PAGE. Kinase dead mutant K85A was used as negative control. (B) Identification of phosphoylation site of Ehactin. The 43 kDa pEhactin band identified in panel (A) was cut out and subjected to mass spectrometry (LC-MS/MS). Table shows summary of the mass spectrometry results, and the peptides that mapped to Ehactin sequence. The CID-MS3 spectrum of the Ehactin VAPEEHPVLLTpEAPMNPK phosphopeptide showed 11^th^ threonine position to be phosphorylated. Prominent y and b ions are shown, and [M + 3H]3+−P = 658. (C) Phosphorylation of rabbit skeletal muscle actin by EhAK1. Rabbit actin (2µg) was incubated with either EhAK1 (2µg) or mutant K85A-EhAK1 (2µg) in presence of kinase assay buffer. The input actin visualized by coomassie staining, is shown in the lower panel. (D) Phosphorylation of rabbit G-actin by EhAK1. G-actin and F-actin were separated by ultracentrifugation (1, 00,000 g) as supernatant (G-actin) and pellet (F-actin). The separated fractions (2µg) were used for kinase assay in the presence of EhAK1. (E) Phosphorylation of rabbit G-actin by EhAK1 in the presence of actin polymerization inhibitor cytochalasin D. (F) Polymerization of phosphorylated rabbit G-actin. G-actin (2µg) was phosphorylated in the presence of EhAK1 (2µg) or K85A (2µg) followed by ultracentrifugation at 1, 00,000 g to separate supernatant (G-actin) and pellet (F-actin). (G) Phosphorylation of Ehactin by EhAK1. *E. histolytica* actin was immunoprecipitated from the total lysate with anti Ehactin antibody. Immunoprecipitated material was used for setting up a kinase reaction with EhAK1. (H) Phosphorylation of GFP-tagged wild type and mutant Ehactin by EhAK1. Over-expressed GFP-tagged wild type or mutant Ehactin was immunoprecipitated from total lysate with anti-GFP antibody. Immunoprecipitated material was used for setting up a kinase reaction with EhAK1.

To identify the possible substrate(s) of EhAK1 we used a mass spectrometric approach, based on identification of *E. histolytica* cytosol proteins phosphorylated by alpha kinase *in vitro* (as described in “Materials and Methods”). Three main phosphorylated bands (50, 43, 35 kDa) were observed in the autoradiogram **(** **Fig. 7A** **)**. Among these, 50 kDa bands is the autophosphorylated form of EhAK1 as it was also seen with the lane containing only the kinase domain. Since the intensity of the 35 kDa band was low, and a faint band at the same position was also visible in the lane containing the kinase dead mutant **(** **Fig. 7A** **)**, we did not pursue this as it may not be a genuine substrate for EhAK1, but phosphorylation product of an endogenous kinase. Moreover, it would have been difficult to identify this protein due to its low amount. Therefore, it is likely that the 43kDa band is the main substrate of EhAK1, though other minor substrate(s) cannot be ruled out. The 43 kDa region of the gel was subjected to mass spectrometry and actin was identified as the main protein **(** **Fig. 7B** **)**. We did not observe peptide fragments from any other protein of 43KDa, though it is likely that the gel fraction may have contained proteins in addition to actin. Peptides were seen from higher molecular weight proteins, such as Enolase (47 kDa). In another approach to identify possible targets, the amoebic proteins binding to EkAK1 were purified by affinity chromatography and the bound proteins were identified by mass spectrometry **(Table S1)**. Actin and actin binding proteins were found to be the major EhAK1 binding partners. Surprisingly we did not observe any of the myosins among the bound proteins, since myosins are known to be likely substrates of alpha kinases. It is possible that myosin may be a substrate of EhAK1 under specific conditions. Actin was further confirmed to be a substrate in an *in vitro* kinase assay in which it was shown to be phosphorylated by the wild type protein but not the kinase dead mutant **(** **Fig. 7C** **)**. Further we performed kinase assay using G-actin and F-actin separately. The results clearly showed that EhAK1 could phosphorylate only G-actin but not F-actin **(** **Fig. 7D** **)**. Phosphorylation of G-actin was tested in the presence of cytochalasin-D, an inhibitor that binds to the barbed end of actin filaments and prevents further polymerization leading to an increase in the availability of G-actin. High level of G-actin phosphorylation was observed on addition of the inhibitor (10 µM) presumably due to enhanced availability of G-actin [27], [46] **(** **Fig. 7E** **)**. Different concentrations of cytochalasin D were tried and it was found that 10 µM was sufficient to obtain maximum effect. When phosphorylated actin was ultracentrifuged, most of the phosphorylated molecules were found in the pellet fraction (F-actin) suggesting that p-G-actin can polymerize into phosphorylated F-actin **(** **Fig. 7F** **)**. No polymerization of G-actin was seen in the presence of K85A-EhAK1 as there was no visible band of radioactive actin in the pellet fraction **(** **Fig. 7F** **)**. Actin was present as non polymerized non radioactive form in the supernatant (coomassie blue stained band) suggesting that phosphorylation of G-actin is linked to its' ability to polymerize to F-actin.

All assays described above have been carried out using rabbit muscle actin as substrate. Therefore, we tested if *E. histolytica* actin can also be used as a substrate by EhAK1. As in the case of rabbit actin, *E. histolytica* actin was also phosphophorylated by wild type EhAK1 but not by K85A-EhAK1 mutant **(** **Fig. 7G** **)**. Further we identified phosphorylation sites in Ehactin by mass spectrometry after immunoprecipitation from lysate using anti actin antibodies followed by phosphorylation by EhAK1 **(** **Fig. 7B** **and Fig. S10)**. Only peptides from actin were observed. Our analysis suggested that Thr 107 is likely to be the main phosphorylation site of *E. histolytica* actin as phosphorylated peptide containing T107 was observed in two independent experiments. We have also observed non phosphorylated peptide containing T107. Although existence of additional phosphorylation sites cannot be ruled out based on protein coverage obtained in this set of experiments, mutation of Threonine 107 to alanine by site directed mutagenesis also confirmed that it is the major phosphorylation site in Ehactin as no phosphorylation of mutant GFP-Ehactin (T107A) was found in presence of EhAK1 **(** **Fig. 7H** **)**. These results suggest that G-actin is a substrate of EhAK1 and Thr 107 is likely to be the main phosphorylation site.

In order to understand the effect of phosphorylation on actin function we analysed sequence conservation and structural features around T107 in both rabbit and amoebic actin **(Fig. S11)**. Sequence alignment clearly showed that T107 and surrounding sequences in both rabbit and *E. histolytica* actin are conserved. The site is located outside helical domains and maps within a beta sheet. Further, we modelled the 3-D structure of wild type Ehactin using structure of *D. discoideum* actin (PDB 3Ci51.A) as a template and found there was a structural difference with repeat to rabbit actin (RSMD value 2.12). The major difference between the two structures was in the unstructured loop regions, while the structure around T107 was found to be conserved **(Fig. S12A, and inset)**. We also modelled T107A mutant Ehactin using *D. discoideum* actin (PDB 3Ci51.A) as a template and did not find any significant difference with wild type Ehactin on superposition (RMSD value of 0.001) **(Fig. S12B)**.

As EhAK1 phosphorylates G-actin and interacts with EhCaBP1, so we further checked whether the presence of EhCaBP1 had any effect on the level of actin phosphorylation. For this we did *in vitro* actin phosphorylation in presence and absence of EhCaBP1 and found that EhCaBP1 binding did not alter the kinase activity of EhAK1 **(Fig. S13)**.

### Actin phosphorylation during erythrophagocytosis

To demonstrate EhAK1-dependent actin phosphorylation *in vivo* we used custom generated phospho-specific antibody (pT107). pT107 recognized only phosphorylated immunoprecipitated GFP-Ehactin but not GFP-T107A actin in western blots. Moreover, phosphatase treatment abolished the signal, suggesting that the antibody was specific for phosphorylated T107 actin **(Fig. S14A)**. This also strengthens our conclusion that T107 is the site of phosphorylation and not another site whose phosphorylation is regulated by T107. This antibody stained actin present at the phagocytic cups suggesting that at least some of this actin may already be phosphorylated **(Fig. S14B)**. Moreover, phosphorylated rabbit actin was also recognized by this antibody suggesting a conservation of T107 phosphorylation site in both *E. histolytica* and rabbit actin **(Fig. S15)**.

**Figure 8:**
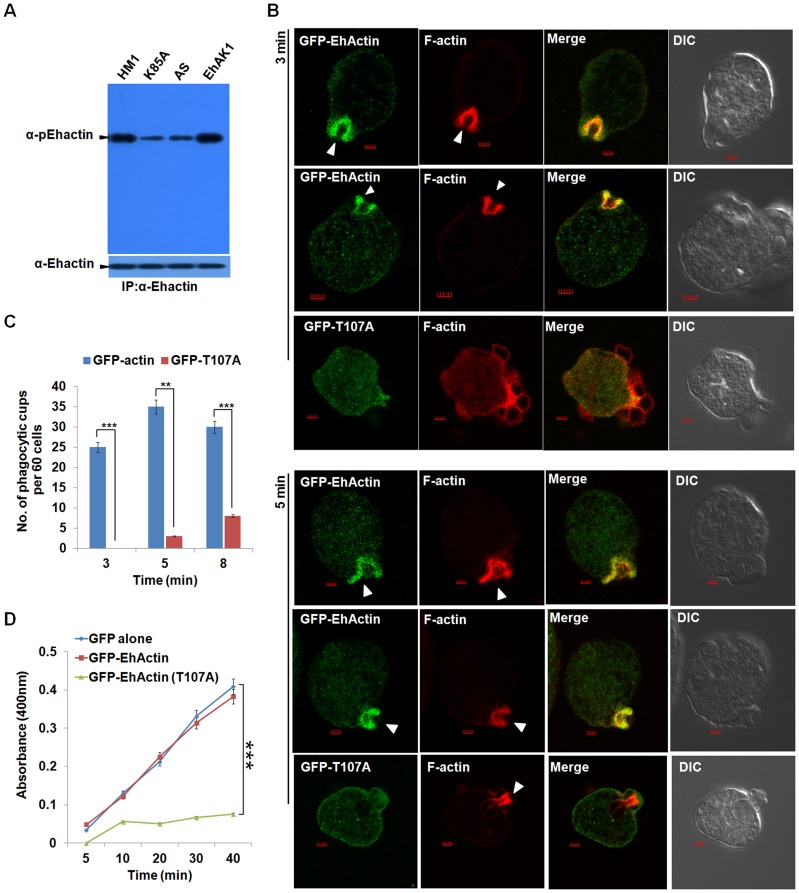
Phosphorylated actin participates in phagocytosis. (A) *E. histolytica* cells carrying EhAK1 construct in the sense and the antisense orientations and K85A in the sense orientation as indicated were induced with tet. Phosphorylated actin was detected by immunoprecipitation using anti Ehactin antibody followed by western blots using anti-pEhactin antibody. (B) Indicated cell lines grown with 30 µg/ml G418 to enhance the expression of transfected gene were incubated with RBC for 3 min at 37°C. The cells were then fixed and immunostained with anti-GFP. F-actin was stained with TRITC-phalloidin. Arrowheads indicate phagocytic cups. Bar represents 20pixels. A few representative cells are shown. (C) Quantitative analysis was carried out by selecting randomly sixty cells from each experiment and the numbers of phagocytic cups present in all cells were counted (blue, GFP-actin; red, GFP-T107A). (D) RBC uptake assay was performed using indicated cells grown with G418. The experiments were carried out three times independently in triplicates. *p-value≤0.05, **p-value≤0.005, ***p-value≤0.0005.

Phospho-specific antibody was used to measure p-actin levels in whole cells containing both G- and F-actin. Antibody against total actin (ICN Biochemicals) was used to determine levels of total actin. There was no significant increase in the level of p-actin in cells over-expressing wild type EhAK1 as compared to untransfected cells (HM1 lane) **(** **Fig. 8A** **)**. In cells expressing K85A-EhAK1 and EhAK1-AS, there was a reduction in p-actin by about 50% and 48% respectively **(** **Fig. 8A** **)**. On treatment of cells with general kinase inhibitors, staurosporine and genistein there was no significant reduction in p-actin levels. Insensitivity to staurosporine and genistein is a known property of alpha kinases [47] **(Fig. S16)**. However, these inhibitors could block autophosphorylation of EhC2PK, a kinase known to be sensitive to these inhibitors [48] **(Fig. S16)**. These results suggest that Thr107 is the major site of actin phosphorylation by EhAK1 *in vivo*. It is unlikely that actin is phosphorylated at this site by any other kinase.

To study the role of phosphorylation in actin mobilisation and dynamics we over-expressed the GFP-tagged wild type and T107A mutant actin by increasing the concentration of G418 as seen by western blot. At 30µg of G418 the mutant actin was present at 2 to 2.5 fold higher concentration than wild type endogenous protein **(Fig. S17)**. While the wild type GFP-Ehactin reached phagocytic cups as rapidly as the endogenous protein, T107A actin showed a defect. Cells expressing high level of this mutant actin displayed reduced phagocytic cup formation at 3 min, with reduced enrichment of T107A actin at the cups. The cells had many attached but not phagocytosed RBCs **(** **Fig. 8B** **)**. Phalloidin staining at the attachment site was also much reduced in these cells compared with cells over-expressing wild type actin, suggesting that the over-expression of mutant actin interfered with recruitment of endogenous actin. By 5 min F-actin levels at some phagocytic sites increased significantly though mutant actin molecules continued to be scarce. Reduced accumulation of endogenous actin in cells over-expressing the mutant actin reflected in the small number of cups formed and the low level of overall phagocytic events in these cells **(** **Fig. 8B** **)**. The number of cups in control cells was 1.5 fold higher than in cells expressing mutant actin at 5 min **(** **Fig. 8C** **)**. Erythrophagocytosis in cells over-expressing T107A was only 18% of control, while there was no significant change in cells over-expressing wild type actin **(** **Fig. 8D** **)**. Our results suggest that phosphorylation of actin by EhAK1 is an important component of actin dynamics in *E. histolytica* in relation to erythrophagocytosis.

### Modulation of actin dynamics by EhAK1

#### Effect of EhAK1 on the amount of F-actin in E. histolytica cells

**Figure 9:**
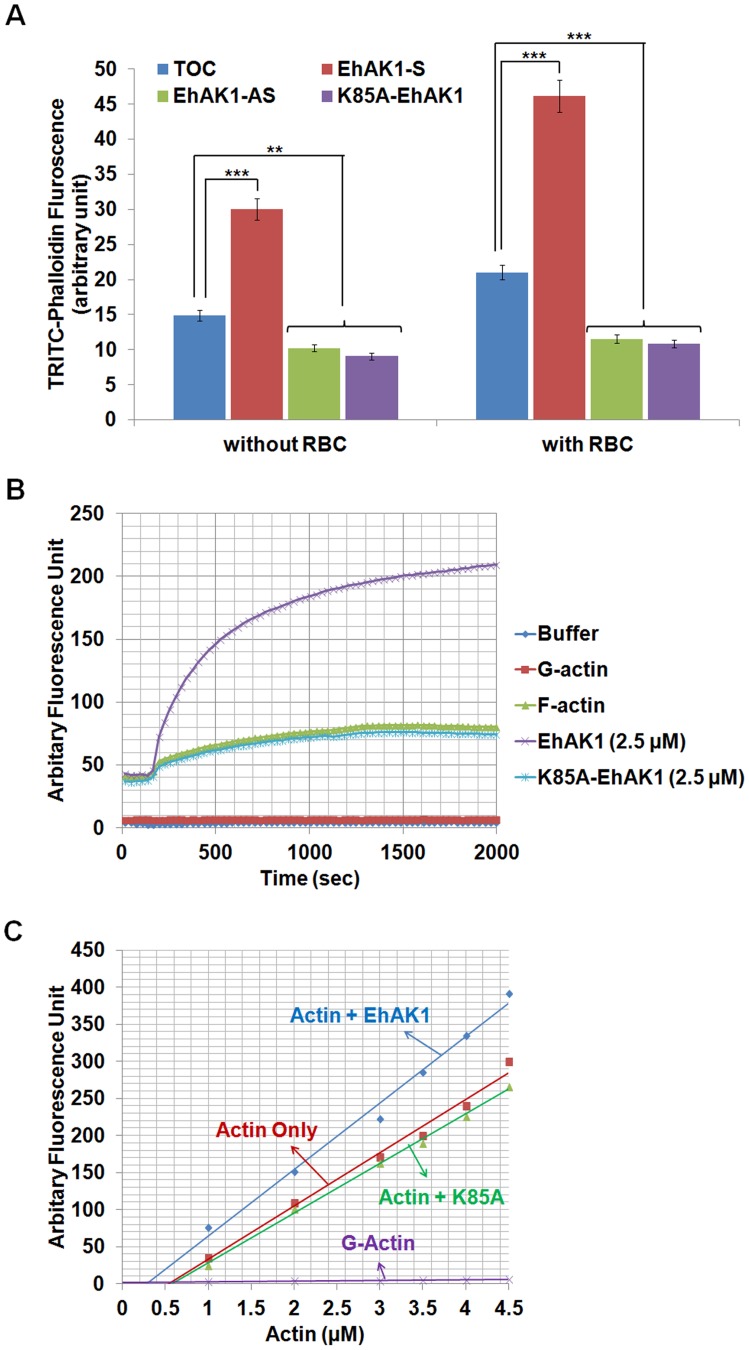
Modulation of actin dynamics by EhAK1. (A) Effect of EhAK1 on F-actin content of *E. histolytica* cells. *E. histolytica* cells (2×10^5^) containing indicated constructs were grown in presence or absence of 30µg/ml tet or G418 for 48 h. Amount of F-actin was measured using TRITC-phalloidin as described in “Materials and Methods”. For phagocytosis, amoebic cells were incubated with RBCs for 10 min at 37°C. (B) Effect of EhAK1 on actin polymerization. Rabbit muscle actin (10∶1 unlabelled and pyrene-labelled actin) was polymerized with and without indicated proteins as described in “Materials and Methods”. The increase in fluorescence of pyrene was observed at 407 nm. (C) Critical concentration was measured as described in “Materials and Methods”. The steady state pyrene fluorescence was recorded after incubating actin as above at the indicated concentration for 16 h at 25°C in the presence of EhAK1 and K85A. *p-value≤0.05, **p-value≤0.005, ***p-value≤0.0005.

In order to see if overall F-actin content is dependent on cellular EhAK1 levels the amount of F-actin in *E. histolytica* cells was measured using a rhodamine-phalloidin based fluorescence assay (see “Materials and Methods”). Over expression of EhAK1 in amoebic cells increased F-actin content by 2.1 fold as compared to cells containing the vector alone. It decreased on expressing antisense EhAK1 and K85A-EhAK1 by 0.7 and 0.6 fold respectively **(** **Fig. 9A** **)**. F-actin content also increased in *E. histolytica* cells undergoing erythrophagocytosis. Comparatively, there was a more pronounced effect observed in EhAK1 over expressing cells than that in cells expressing K85A-EhAK1 or antisense RNA **(** **Fig. 9A** **)**. This could be due to a number of factors, such as other proteins regulating actin dynamics and our determination of F-actin levels in whole cells rather than in localized areas. These results indicate a correlation between F-actin content and cellular level of EhAK1.

#### EhAK1 enhanced the rate of actin polymerization by phosphorylation of G-actin

In a previous section we have shown that phosphorylated G-actin is efficiently polymerized to F-actin. We checked the rate of actin polymerization in the presence of EhAK1 and found that it increased by 5±0.1 fold (0.25 µMS^−1^) compared with the rate of polymerization in the absence of EhAK1 (0.05 µMS^−1^) or in the presence of kinase dead mutant (0.048 µMS^−1^) **(** **Fig. 9B** **)**. These results show that phosphorylation by EhAK1 increases the rate of actin polymerization. This is probably achieved by altering the critical concentration of actin required for polymerization. To test this, different concentrations of actin were taken for polymerization (overnight at 25°C) in the presence and the absence of EhAK1. The results showed that EhAK1 lowered the critical concentration for polymerization from 0.55 µM to 0.28 µM **(** **Fig. 9C** **)**. K85A-EhAK1 did not change the critical concentration (0.58 µM) **(** **Fig. 9C** **)**. Therefore, it appears that phosphorylation has an important role in Ehactin dynamics.

## Discussion

The spatial and temporal regulation of localized actin dynamics plays a central role in cellular processes, such as phagocytosis and psuedopod formation [49]. Actin cytoskeleton complex is assembled at the site of phagocytosis by recruitment of a number of components that help in the assembly of actin filaments which generate the force required for engulfing the particles being phagocytosed. The structure is then disassembled so that the components can be used elsewhere. In highly phagocytosing cells, such as *E. histolytica* the entire cycle must occur rapidly so that the cells are able to phagocytose a large number of particles in a short period [50]. We have been investigating the molecular mechanism by which signalling is relayed in erythrophagocytosis from the initiation site at the membrane, leading to formation of a dynamic actin cytoskeleton complex. Since mechanisms of phagocytosis vary depending upon the particle (live or apoptotic mammalian cells, bacteria, RBC) used we have restricted our study to phagocytosis of RBCs [13].

In this manuscript we show that an alpha kinase EhAK1 is recruited at the initiation site and is directly involved in generating dynamic actin filaments. The results presented here suggest that EhAK1 is recruited to the phagocytic cups through EhCaBP1 bound to membrane associated EhC2PK [28]. This recruitment is specific as EhAK1 does not bind EhCaBP3 [29]. Since EhCaBP1 forms a trimeric structure, it is possible that it can form a complex with multiple binding proteins, for example EhC2PK and EhAK1 [51]. The formation of a protein-complex may help in stabilizing phagocytic cups and their progression towards phagosome formation. The binding of EhAK1 to EhCaBP1 takes place only in the presence of Ca^2+^, which is different from the binding of EhCaBP1 to EhC2PK where Ca^2+^ is not required. Thus EhCaBP1 binds to its interacting partners in multiple ways, and Ca^2+^ plays a subtle role by manipulating the interaction of some of the components, which may determine their specific function in phagocytosis.

Two main lines of evidence suggest that EhAK1 is involved in phagocytosis; its presence at phagocytic cups during phagosome formation, and reduction in the formation of phagocytic cups on its down-regulation. Concentration of EhAK1 appears to directly influence the rate of phagocytosis. It is likely that a threshold concentration of EhAK1 is needed to initiate the process. Since some amount of protein is present even on down-regulation by antisense RNA, phagocytosis continues to take place, but at a slow pace as the time taken by the molecule to reach the critical level would be longer. Conversely, over-expression of EhAK1 resulted in an increase in phagocytosis. Thus the rate of formation and progression of phagocytic cups may be directly proportional to the concentration of some key molecules, such as EhAK1 and its' recruitment at the phagocytic cups. More data are required to validate this further. Interestingly EhAK1 has an SH3 domain in addition to an alpha kinase domain. Our results suggest that the kinase but not SH3 domain is involved in EhCaBP1 binding. In fact it appears that SH3 domain may have a negative effect, since we observed low level of Ca^2+^-independent binding of EhCaBP1 to KD, but no binding was observed with full length EhAK1. This was a surprising observation as SH3 domains are known to participate in protein-protein interaction and in recruitment at signalling sites [52]. We also cannot rule out at this stage the role of SH3 domain in recruitment of accessory proteins in phagosome formation.

The results presented here clearly show that actin is one of the substrates of EhAK1, although alpha kinases have so far been reported to mainly phosphorylate myosin [38]. It is unlikely that myosins are one of the substrates as these were not only seen in original screen, but also were not observed when EhAK1 affinity chromatography was used for identifying its' binding partners. Though we find actin as a major substrate, other substrate(s) of EhAK1 cannot be ruled. We have identified only one phosphorylation site in Ehactin. It is possible that additional phosphorylation sites exist but were not seen due to partial (41%) coverage in mass-spectrometry. However, this appears unlikely as no significant phosphorylation was observed in the T107A actin mutant in presence of EhAK1. Moreover, results obtained with pT107 antibody clearly show importance of T107 phosphorylation in phagocytosis. Interestingly T107 maps outside alpha helical regions of actin. The site is not only conserved in both rabbit and amoebic actins, but also the 3D structures are nearly identical, with RMSD 2.12. Therefore, it is not surprising that EhAK1 also phosphorylates rabbit actin and the phosphorylated rabbit actin is recognized by actin pT107. Some alpha kinases (for example, eEF2 kinase) are also known to phosphorylate non alpha helical regions. Moreover, TRPM6 and TRPM7 also phosphorylate MHCB and MHCC, but unlike myosin heavy chain kinases of *Dictyostelium*, non helical regions are phosphorylated [53]–[54]. Therefore, it is not surprising that EhAK1 phosphorylate actin at a non helical region. Our data suggest that phosphorylation of actin by EhAK1 helps phagocytic cups to become phagosomes through enhanced actin dynamics. The evidence for the latter comes from over-expression studies using T107A actin mutant cell-line where a dominant negative phenotype was observed with respect to phagocytosis. This is possible if phosphorylated actin has a major role and is also preferred in formation of F-actin network, particularly during phagocytosis. This was seen in experiments done both *in vitro* and *in vivo*. The content of F-actin, initiation and progression of phagocytosis were reduced in cells over-expressing T107A actin, and the *in vitro* rate of actin polymerization was also impaired in the mutant. Quantitative discrepancy between results from *in vitro* and *in vivo* experiments may be due to a difference in behaviour of rabbit and amoebic actins.

During progression of phagocytic cups towards phagosome, EhAK1 is mainly present at the advancing end of the cup, and not so much at the base of the cup. This is similar to Ptdins(4,5)P2, GTP-CDC-42 and GTP-ARF6 [55]. All of these molecules are associated with phagocytosis and their presence at the tip suggests their participation in localized actin dynamics required to push psuedopods to engulf RBCs. Though phosphorylation of actin has been observed in many different systems, it was not observed in *E. histolytica* prior to this report. A cAMP-dependent protein kinase was shown to phosphorylate chicken smooth muscle G-actin [56]. In *Amoeba proteus* also only G-actin was found to be phosphorylated, but the phosphorylated actin was not able to polymerize [57]. An atypical kinase, actin fragmin kinase of *Physarum polycephalum* [58] was shown to phosphorylate the T202 residue of G-actin, and on phosphorylation filament formation was blocked. In *D. discoideum* Tyr-53 residue of actin gets phosphorylated [59] and affects formation of actin filaments. The properties of phosphorylated actin of *E. histolytica* are different from other examples except that EhAK1 also phosphorylates only G-actin and not F-actin. In *E. histolytica*, the phosphorylated form of G-actin displays enhanced capability to form filaments. We are still not clear about the mechanism of enhanced polymerization of G-actin on phosphorylation.

Our earlier studies on EhCaBP1 revealed that cell proliferation gets affected on either expression blocking or over expression of Ca^2+^ binding defective mutant of this protein [27], [39]. Though it appears that this may be due to its' effect on phagocytosis and macropinocytosis as inhibition in these processes may reduce nutrient uptake, participation of other pathways cannot be ruled out. Since EhAK1 is a downstream signal transduction molecule of EhCaBP1, it is not surprising to observe a similar effect on cell proliferation on either down regulation of EhAK1 expression or over expression of a dominant negative mutant. We are also currently attempting to identify non phagocytic pathways where EhCaBP1-EhAK1 system may participate.

**Figure 10:**
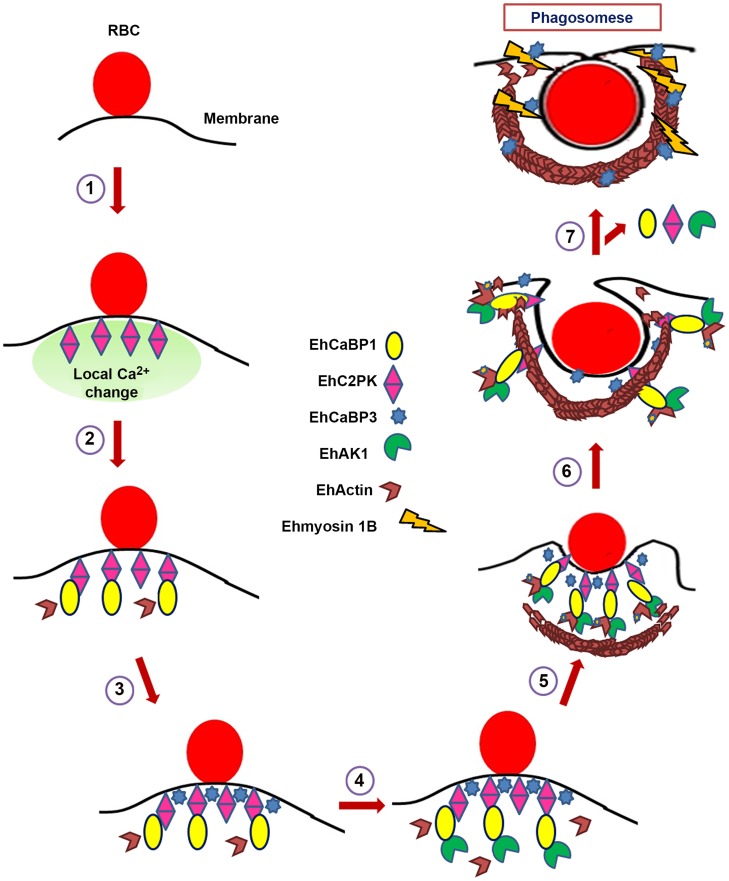
Model depicting RBCs phagocytosis in *E. histolytica*. Model summarises predicted course of events for initiation of phagocytosis in *E. histolytica* as derived from our experiments. There are other molecules involved in the process, but these are not shown here, for brevity. 1. Attachment of RBC to the membrane increases concentration of EhC2PK at the attachment site (Ca^2+^ dependent). 2. Recruitment of EhCaBP1 at the site through interaction with EhC2PK (Ca^2+^ independent). Recruitment of some actin molecules. 3. Recruitment of EhCaBP3 at the site (independent of EhCaBP1). Recruitment of some actin molecules. 4. Recruitment of EhAK1 at the site through binding with EhCaBP1 (Ca^2+^ dependent). 5. Recruitment of other actin modulating molecules. Activation of actin dynamics pathway including phosphorylation by EhAK1 leading to enhanced actin dynamics. 6. Actin polymerization and progression of cups towards phagosome. 7. Recruitment of Ehmyosin 1B through EhCaBP3 and EhCaBP1, EhC2PK, EhAK1 leave.

In conclusion, we have described a novel mechanism of regulation of phagocytosis in *E. histolytica* through manipulation of actin dynamics. The proposed model based on this and our previous work is shown in Fig. 10. The pathway initiated by accumulation of EhC2PK after binding of RBC and recruitment of EhCaBP1. This pathway may also be involved in other processes, such as trogocytosis that is likely to be one of the mechanisms of tissue invasion [60]. The atypical kinase EhAK1 is recruited to the phagocytic cups through interaction with EhCaBP1. Phosphorylation of localized G-actin leads to increased rate of polymerization of actin at the tip of the expanding cup, eventually engulfing the particle. Overall the coupling of actin dynamics to signalling events initiated on attachment of RBC is the key to successful completion of phagocytosis. There are a number of unknown aspects of the pathway simplistically depicted in the figure. We still do not know the substrate(s) of EhC2PK; actin is definitely not a substrate (unpublished data). However, the kinase activity is required for propagation of the signal leading to phagosome formation. We believe that EhCaBP3 recruitment and function is independent of EhCaBP1. Its' major role is to further recruit myosin1B and help in cessation process. We also do not know the functions of SH3 domain and other alpha kinases encoded by the genome. We do plan to systemically investigate some of these questions in future. Our studies carried out so far including results presented in this report suggest that *E. histolytica* displays a novel signal initiation pathway that involves participation of calcium binding proteins and uses EhAK1 as one of the main molecules that couple the two process, signalling and actin dynamics. *E. histolytica* being an early branching eukaryote, deciphering molecular mechanisms of phagocytosis in this organism will help us to understand the evolution of related pathways and processes in different systems.

## Materials and Methods

### Ethics statement

Both mice and rabbits used for generation of antibodies were approved by the Institutional Animal Ethics Committee (IAEC), Jawaharlal Nehru University (IAEC Code No. : 18/2010). All animal experimentations were performed according to the National Regulatory Guidelines issued by CPSEA (Committee for the Purpose of Supervision of Experiments on Animals), Ministry of Environment and Forest, Govt. of India.

### Growth, maintenance, antibiotics and transfection of *E. histolytica*

*E. histolytica* strain HM-1 trophozoites were maintained and grown in TYI-S-33 medium supplemented with 125 µl of 250 U/ml penicillin G (potassium salt from Sigma) and 0.25 mg/ml streptomycin per 100 ml of medium as described before [61]. *E. histolytica* trophozoites were transfected following methods described before [27]. The transformants containing tetracycline inducible system and constitutive GFP expression system were grown in the presence of 10 µg/ml of hygromycin B and 10 µg/ml of G418, respectively. For individual experiments, transfected cells were first grown for 24 h (60–70% confluent) and then induced by adding 30 µg/ml tetracycline in tetracycline inducible system and 30 µg/ml of G418 for constitutive GFP expression system respectively for 48 h.

### Cloning of various constructs used in this study

EhAK1 gene was cloned in the shuttle vector pEhHYG-tetR-O-CAT in place of CAT gene using KpnI and BamHI in either the sense or the antisense orientation. The full-length gene EhAK1, Kinase domain (KD), K85A and Ehactin wild type and mutant T107A were cloned in pEh-Neo-GFP vector at Xho 1 and BamH1 sites such that GFP tags are at the amino terminal end of indicated proteins. Kinase dead mutant (K85A) and Ehactin T107A were made by site-directed mutagenesis. Oligonucleotides used for making the above stated constructs are described below.

EhAK1F-5′CGCGAATTCGTCACGGAGATATGATGGTAGAT, EhAK1R-5′CGCGTC GACCATTGTAACA GTTTGTACTTTTG, KD F-5′CGCCATGGGCTCACGAGATATG ATGGTAGAT, KD R-5′ CGCCTCGAGTGTAGTACCTTTTGTAGTGTCTG, Antisense F-5′CGCGGATCCTCACGAGATATGATGGTAGATCC, Antisense R5′CCCGGTACC TTACATTGTAACAGTTTGTACTTTTGC, Sense F-5′CCCGGTACCTCACGAGATAT GATGGTAGATCC, Sense R-5′CGGGGATCCTTACATTGTAACAGTTTGTACTTTT GC, EhAK1 GFP F-5′ CCCCTCGAGTCACGAGATATGATGGTAGATCC, KD GFP F-5′CCCCTCGAGTCACGAGATATGATGGTAGATCC, Actin GFP F -5′GGCCCTCG AGGGAGACGAAGAAGTTCAAGC, Actin GFP R 5′ CCGGGGATCCTTAGAAGCAT TTTCTGTGGAC.

### Primers for site directed mutagenesis

K85AF 5′CGTGGAGACAGAATTGTTCTTGCACGATTTTTCCAACAAAGACCGC K85AR 5′GCGGTCTTTGTTGGAAAAATCGTGCAAGAACAATTCTGTCTCCACG R69AF 5′ CCATTTGCAAAAGGAGGAGAAGCTCTTGCTTTTCGTGC R69A R 5′ GCACGAAAAGCAAGAGCTTCTCCTCCTTTTGCAAATGG D223A F 5′CAATAATAAATTTTATCTTACTGCTCCAGCATTACATCA D223A R 5′CTATATGATGTAATGCTGGAGCAGTAAGATAAAATTTAT Actin T107A F 5′GAACATCCAGTTCTTTTAGCTGAAGCCCCAATGAATCC Actin T107A R 5′GGATTCATTGGGGCTTCAGCTAAAAGAACTGGATGTTC

### Immunoprecipitation

Cell lysate for immunoprecipitation contained 10 mM Tris-HCl, pH 7.5, 150 mM NaCl, 2 mM p-hydroxymercuribenzoic acid (PHMB), 1 mM phenylmethylsulfonyl fluoride (PMSF), protease inhibitor cocktail, 2 mM β-ME and 1% Triton ×100 and was prepared as described before [27]. It was used after centrifugation at 15,000 rpm for removing cellular debris. Anti-EhAK1 antibody was conjugated to CNBr-activated Sepharose (1 g, Pharmacia) that was activated and processed as per the manufacturer's protocol. The conjugated CNBr-Sepharose beads were incubated with *E. histolytica* lysate (500µg) for 4 h at 4°C. The beads were then washed with wash buffer (10 mM Tris-Cl (pH 7.5), 150 mM NaCl, 1 mM imidazole, 1 mM magnesium acetate, 2 mM β-ME, 0.1% Triton ×100 and protease inhibitor cocktail) thrice. Ca^2+^ is present unless otherwise indicated. Beads were washed with 0.06 mM Tris-Cl (pH 6.8) and 100 mM NaCl and finally with 0.06 mM Tris-Cl (pH 6.8). The pellet was suspended in 2× SDS polyacrylamide gel electrophoresis (PAGE) buffer and boiled for 5 min followed by centrifugation for 5 min. The proteins were then analysed by western blotting. For immunoprecipitation of Ehactin, 5 µl of anti-Ehactin antibodies (monoclonal, ICN Biochemicals) at 1∶1000 dilution was incubated with pre-cleared amoebic lysate and then allowed to bind with protein A-Sepharose beads (Amersham) for 2 h at 4°C as described before ([27]. Thereafter we followed the same protocol as described above.

### Western blotting

For immunodetection, samples were separated on 10–12% SDS–PAGE as required. The gel was then transferred on to a polyvinylidine fluoride membrane (PVDF) using a semi dry transfer system and was further processed following standard methods. The antigens were detected with polyclonal antibodies raised in rabbit or mice as indicated (Anti-EhCaBP1 and EhCaBP3; 1∶5000, Anti-pEhC2PK; 1∶100) anti-pEhActin (1∶100 custom made from Abmart, china) followed by secondary anti-rabbit and anti-mice immunoglobulins conjugated to HRPO (1∶10,000, Sigma). ECL reagents were used for visualization (Millipore). GFP and GST antibodies used were obtained from Molecular probes and Santa Cruz, respectively. The concentration of proteins in a sample was estimated by bicinchoninic acid assay using BSA as a standard.

### GST-bead pull down assay

Purified GST-EhAK1 was allowed to bind Glutathione beads (Amersham) for 1 h at 4°C in 1% PBS/BSA/0.1% tween-20. EhCaBP1 or EhCaBP1ΔEF was then added to the reaction and the reaction was incubated for 2 h at 4°C. The beads were then washed thrice with 1% PBS/BSA/0.1% tween-20, and twice with PBS. Bound proteins, eluted by adding 2× SDS–PAGE buffer, were analysed by western blotting. The same procedure was followed for other proteins with GST-tags.

### Immunofluorescence staining

Immunostaining was carried out as described before [27]. Briefly *E. histolytica* cells were harvested via centrifugation and washed with phosphosaline buffer and re-suspended in TYI-33 medium. The cells were then transferred onto acetone-cleaned coverslips placed in a petri dish and was allowed to adhere for 10 min at 35.5°C. The culture medium was removed and the cells were fixed with 3.7% pre-warmed paraformaldehyde for 30 min. After fixation, the cells were permeabilized with 0.1% Triton X-100/PBS for 1 min. The fixed cells were then washed with PBS and quenched for 30 min in PBS containing 50 mM NH_4_Cl. The coverslips were blocked with 1% BSA/PBS for 30 min, followed by incubation with primary antibody at 37°C for 1 h. The coverslips were washed thrice with PBS followed by 1% BSA/PBS before incubation with secondary antibody for 30 min at 37°C. Antibody dilutions used were: anti-EhAK1 at 1∶100, anti-EhCaBP1 at 1∶200, anti-EhCaBP3 at 1∶200, 1∶300 dilution of anti-rabbit Alexa 488, 556 and anti-mice Alexa556 (Molecular Probes). TRITC-Phalloidin was used at 1∶250. The preparations were further washed with PBS and mounted on a glass slide using DABCO (1,4-diazbicyclo (2,2,2) octane (Sigma) 2.5% in 80% glycerol). The edges of the coverslips were sealed with nail-paint to avoid drying. Confocal images were visualized using an Olympus FLUOVIEW FV1000 laser scanning microscope with objective lenses PLAPON 60× O, NA- 1.42. The raw images were processed using FV10-ASW 1.7 viewer or Image J software. Colocalization analysis was done by using JACoP (Image J).

### Fluorescent labelling of RBCs

RBCs were stained with CFSE (Carboxyfluorescein succinimidyl ester) following a modified protocol (Cell Trace CFSE proliferation kit, Invitrogen). Cells (2×10^7^ cells/ml) were incubated in CFSE staining buffer (PBS containing 0.1% BSA and 2.5 µM CFSE) for 10 min at 37°C. The reaction was blocked with complete medium in presence of 2% serum for 10 min on ice, after which, RBC were washed three times with an excess of incomplete media of *E. histolytica*.

### Time-lapse imaging

The cells expressing GFP-EhAK1 were plated onto a 35 mm glass bottom dish with 20 mm bottom well (In Vitro Scientific) and then allowed to settle down and attached to the plate. The dish was kept on a platform with a temperature controller to maintain temperature at 37°C. High-resolution fluorescent time-lapse imaging (Nikon A1R, Optics- Plan Apo VC60× oil DIC N2, Camera- Nikon A1, NA-1.4, RI-1.515) of a moving and phagocytosing amoeba was performed. The images were captured at 3s interval. The raw images were processed using NIS element 3.20 or Image J software available freely on the web (http://rsb.info.nih.gov/ij/).

### Phagocytosis of red blood cells by *E. histolytica* trophozoites

*E. histolytica* trophozoites were harvested in phosphosaline buffer and equal numbers of amoebic cells (10^5^ cells) were incubated with ten million RBCs, previously washed with PBS and incomplete TYI-33 for varying times at 37°C. Amoebae and erythrocytes were collected by centrifugation and non-engulfed RBCs were lysed with cold distilled water and centrifuged at 1000 g for 2 min. This step was repeated twice, followed by resuspending the pellet in 1 ml formic acid to burst amoebae containing engulfed RBCs. The optical density of the samples was determined by spectrophotometry at 400 nm using formic acid as the blank.

### Actin polymerization assay and determination of critical concentration

Polymerization assay was done as per the protocol supplied by the manufacturer (www.cytoskeleton.com). Briefly, polymerization of actin was monitored by an increase in fluorescence of pyrene-labeled actin (cytoskeleton, USA) with excitation at 366 nm and emission at 407 nm. The assays were carried out in a Cary Eclipse Varian fluorescence spectrophotometer. A 100 µl sample containing 3 µM G-actin (10% pyrene labelled G-actin), was saturated with EhAK1 or K85A-EhAK1 at 2.5 µM and the reactions were carried out in polymerization buffer (5 mM Tris-HCl, pH 7.5, 1 mM dithiothreitol, 0.1 mM CaCl_2_, 0.01% NaN_3_, 100 m M KCl and 2 mM MgCl_2_, 1 mM ATP). For critical concentration curve, 10 µM of G-Actin (10% pyrene labelled) was polymerized in polymerization buffer and was then diluted in the same buffer to various concentrations (1, 2, 3, 4 and 5 µM). Before recording the steady state fluorescence, diluted actin was incubated for 16 h at 25°C in the presence or the absence of EhAK1 (2.5µM) and K85A (2.5µM).

### Quantitation of polymerized actin

F-actin was quantified by using TRITC–phalloidin staining as described [62]. Indicated amoebic cells were grown for 48 h in the presence of 30µg/ml tetracycline and 30µg/ml G418 respectively. The cells were harvested and washed with cold phosphosaline buffer. The cells were resuspended in PBS # 7 and 2×10^5^ cells were aliquoted in triplicate and incubated for 10 min at 37°C. The cells were then treated for 1 min with 1% (v/v) triton-X100, and cells were collected and washed three times with PBS # 7. Staining was carried out by adding TRITC-phalloidin (3µM) for 1 h at room temperature on a shaker to keep the cells suspended. Thereafter, the cells were centrifuged at 12,000 g for 2 min, to remove unbound TRITC-phalloidin. Pellet was disrupted by shaking and TRITC-phalloidin was extracted for 15 min by adding 1 ml of methanol. After centrifugation at 12,000 g for 30 s, the supernatant fraction was collected and the fluorescence was measured in a Cary Eclipse Varian fluorescence spectrophotometer at excitation and emission wavelengths of 540 nm and 565 nm respectively. For erythrophagocytosis, amoebic cells (1×10^5^) were challenged with RBCs (amoeba: RBC; 1∶100) for 10 min before extraction.

### Sub-cellular fractionation of amoebic extract

#### Total cell lysate preparation

One million trophozoites growing in log phase were harvested at 280 g for 7 min at 4°C. The pellet was washed with cold PBS # 8 and then re-suspended in 10 mM Tris-Cl pH 7.5, 150 mM NaCl, 1% Triton-X100, 2 mM PHMB and 1× protease inhibitor cocktail (Sigma). Lysate was prepared by first freeze thawing three times followed by sonication for 10 s to shear the genomic DNA and centrifugation at 13000 g for 5 min to pellet down the debris. The supernatant was collected and labelled as total cell lysate.

#### Sub-cellular fractionation

To separate membrane proteins from cytoplasmic fraction, the cell extract was prepared by re-suspending the cell pellet (∼10^7^, washed with PBS # 8) in 1 ml of 100 mM NaHPO_4_ buffer containing protease inhibitors (10 mM NEM, 2 mM PMSF, 0.01 mM leupeptin and 2 mM PHMB). The suspension was then subjected to three cycles of freeze-thawing followed by centrifugation at 100,000×g for 30 min at 4°C. The resulting supernatant was labelled as the cytoplasmic fraction and the pellet which contained the membrane fraction was processed further. The pellet was washed twice with above buffer and re-suspended in the same buffer containing 1% Triton-X100 and re centrifuged at 100,000×g for 20 min at 4°C to separate triton soluble fraction from triton insoluble fraction. The protein content of each fraction was estimated by BCA assay.

#### Phospho-proteomics

In phospho-proteomics first we did kinase assay and then LC-MS/MS as described below.

#### Kinase assay

Actin or substrate phosphorylation was measured as the amount of radioactivity incorporated (γ-^32^P-ATP) into the band which co-migrated with purified recombinant protein. We have already seen that the phosphorylated and non phosphorylated forms of actin co-migrate in SDS-PAGE. The standard reaction mixture (40 µl final volume) contained 0.5 mM MgCl_2_, 30 mM HEPES (pH 7.5), protease inhibitor, phosphatase inhibitor cocktail and pure kinase (2 µg). Reactions were initiated by the addition of (γ-^32^P-ATP) (6000 Ci/mmol) to a final concentration of 2.5 µM and incubated at 30°C for 1 h and was stopped by adding SDS sample buffer containing 50 mM EDTA followed by boiling. The samples were than resolved on SDS-PAGE. Radioactive bands were detected by a Phosphor Imager (Fujifilm). For phosphoproteomics we performed kinase assay by adding non-radioactive ATP (sigma) and reaction was resolved on SDS-PAGE. The substrate used was either whole cell lysate or immunoprecipitated actin as indicated.

#### Mass spectrometry

 SDS-PAGE protein band was excised and then subjected to in-gel trypsin digestion. Briefly, the excised gel was sliced to small pieces, transferred to a sterile siliconized tube and destained by repeated washing with 50 mM NH_4_HCO_3_ and 50% acetonitrile. Reduction was carried out by adding 75 µl of 10 mM stock of dithiothreitol and incubation at 55°C for 30 min. The solution was removed and 50 µl of 50 mM iodoacetamide (IAA) was added and the mixture was incubated further at room temperature, in the dark, for 40 min. Enzymatic digestion was carried out by incubating the reaction mixture with trypsin (40 ng/μl of 25 mM NH_4_HCO_3_) overnight at 37°C. Digested peptides were reconstituted in 15µl of the 0.1% formic acid and 3µl of the same was used for standard 70 min gradient RPLC-MS/MS analysis, followed by acquisition of the data on LTQ-Orbitrap-MS using DDNLMS3 (data dependent neutral loss MS3) scanning. Generated data was matched against NCBI, using Sequest search engine on Proteome discoverer 1.3 and checked for phosphorylation. Minimum of two High confident peptides was used as a prerequisite to identify the proteins. Standard phosphopeptide (25fmoles of mix) was spiked into 250 fmoles of Standard BSA digest and analyzed to check the performance of the instrument.

### Affinity purification and identification of EhAK1 binding proteins

 Conjugation of EhAK1 to CNBr-activated Sepharose 4B was done as described before [29]. For purification of EhAK1 binding protein, *E. histolytica* total cell lysate was made as described above. The total cell lysate was loaded into EhAK1- Sepharose column and flow through was passed 3-4 times at a rate of 0.1 ml/min. unbound proteins were washed with 30 ml bed volume of lysis buffer. The bound proteins were eluted with 0.1M glycine, pH 2.5 and immediately 1/10^th^ volume of 1M Tris-cl (pH 8) was added to maintain the pH. The entire chromatographic step was carried at 4°C. The eluted desalted samples were analysed by LC-MS as described above.

### General methods

All SDS-PAGE gel electrophoresis was done using 10% acrylamide unless otherwise indicated. Proteins were estimated by BCA assay and we have standard protocols were used for all molecular techniques.

### Statistical analysis

Statistical comparisons were made using a one-way ANOVA test. Experimental values were reported as the means ± s.e. Differences in mean values were considered significant at *p-value≤0.05, **p-value≤0.005, ***p-value≤0.0005. All calculations of statistical significance were made using the GraphPad InStat software package (GraphPad).

## Supporting Information

Figure S1 **Domain organization and phylogenetic analysis of different alpha kinases encoded by** ***E. histolytica*** **.** (A) Phylogenetic analysis of the alpha kinase family of *E. histolytica*. A PSI-Blast search was done to identify all sequenced alpha kinases from genome database using EhAK1 alpha kinase domain. For some of the predicted sequences that did not display alpha kinase domain using Scan prosite, reciprocal PSI-Blast searches were performed to ensure that they were indeed homologs of alpha kinases. Selected sequences were then aligned using ClustalW2. A phylogenetic tree was generated using PHYML. Bootstrap values of the major branches are indicated. (B) (A) Schematic presentation of domain organization of different alpha kinases EhAK1 (XP_656642), EhAK2 (XP_651695), EhAK3 (XP_652177), EhAK4 (XP_654603), and EhAK5 (XP_654482) encoded by *E. histolytica genome*.
(TIF)

Figure S2 **Multiple sequence alignment of the kinase domain of EhAK1 with respective domains of alpha kinases from** ***D. discoideum.*** *D. discoideum* alpha-kinases (MHCK A-P42527, MHCKB-P90648, MHCKC-Q8MY12, AK1-Q54DK4, VWkA-Q6B9X6) and EhAK1 alpha-kinase domain (C4M9G9) were aligned using ClustalW2 and clustered into eight sub domains. *D. discoideum* (D.d), *E. histolytica* (E.h) and sequences representing P-loop, G-rich region and Zinc finger motif are indicated. Invariant K85 that is predicted to be involved in nucleoside binding site is also shown.
(TIF)

Figure S3 **Purification of recombinant wild type EhAK1 and K85A-EhAK1.** SDS page analysis of purified HIS-tagged wild type EhAK1 and mutant K85A-EhAK1 is shown.
(TIF)

Figure S4 ***In vivo*** **expression of EhAK1.** (A) Western blot analysis was used for checking the specificity of raised Anti-EhAK1 antibody in Entamoeba lysate (Anti-EhAK1 1;1000). Pre-bleed was taken as control. (B) Schematic representation of the constitutive expression system used for expression of GFP-conjugated proteins in amoebic cells. Western blot analysis for detection of endogenous EhAK1, overexpressed GFP, GFP-EhAK1 and GFP-KD. Total cell lysate (50 µg) was separated in SDS-PAGE and were transferred on to a PVDF membrane for immunodetection. Anti-GFP antibody was used at 1∶3000 dilution. The bound antibodies were identified by an appropriate peroxidase-labelled secondary antibody raised against rabbit immunoglobulins and visualized with ECL reagents. (C) and (D) Immunolocalization of EhAK1 in indicated *E. histolytica* cells. Transfectants containing GFP-EhAK1, GFP-KD and only GFP vector or normal amoebic cells were grown in presence or absence of 30µg/ml G418. Immunofluorescence was performed using anti-EhAK1 and anti-GFP antibodies followed by Alexa-555 (red) and Alexa-488 (green) or Pacific blue-410 respectively. Nucleus was stained using Hoechst (Blue). (Scale bar, 5 µm; DIC, differential interference contrast). Quantitative analysis of fluorescent signals was done as in Fig. 1d. (E) Imaging of EhAK1 during erythrophagocytosis in cells containing GFP or GFP-EhAK1 constructs. Cells were grown for 48 h and incubated with RBC for 5 min at 37°C. Immunostaining was performed using anti-GFP or anti-EhAK1 antibodies followed by Pacific blue-410 and Alexa-488 respectively. F-actin was stained with TRITC-phalloidin. Arrowheads indicate phagocytic cups. Quantitative analysis of fluorescent signals was done as in Fig. 1d. Bar represents 5 µm.
(TIF)

Figure S5 **Over-expression of antisense RNA of EhAK1.** Imaging of EhAK1 in *E. histolytica* cells. Normal amoeba and EhAK1-AS were grown for 48 h in presence or absence of 20 µg/ml tet. Immunostaining was performed using anti-EhAK1 antibody followed by Alexa-488. F-actin was stained with TRITC-phalloidin. Quantitative analysis of fluorescent signals obtained by immunostaining of EhAK1 from different locations in *E. histolytica* cells and EhAK1 transfectants. For analysis, five random regions were selected and average intensity was computed for each region. This was repeated for five such cells (N = 5, bars represent standard error). Bar represents 10 µm.
(TIF)

Figure S6 **Phagocytic uptake of fluorescent labelled RBCs.** (A) Amoebic cells with and without indicated constructs were incubated with fluorescent labelled RBCs for indicated time at 37°C. These cells were then fixed and stained with TRITC-Phalloidin. Arrows show attached RBCs at the site of phagocytosis and star marks the phagocytized RBCs. (B) Z-section of the amoebic cells incubated with fluorescent RBC's (Green) and stained with TRITC-phalloidin (Red). (C) Quantitative analysis was carried out by selecting randomly fifty cells from each experiment and the numbers of phagocytized RBCs present in all cells were counted.
(TIF)

Figure S7 **Proliferation of** ***E. histolytica*** **cells in presence of different constructs.** All cells were grown in presence of 20 µg/ml hygromycin/G418 and tetracycline was added to the medium at 20 µg/ml at 0 h. Cells were grown in 5 ml culture tubes in triplicate for all the experiments and counting was carried out using a haemocytometer, after chilling the tube for 5 min.
(TIF)

Figure S8 ***In vitro*** **competition assay.** *In vitro* competition assay of K85A-EhAK1 mutant and wild type EhAK1. Recombinant wild type EhAK1 (2µg) was incubated with EhCaBP1 (2 µg) in presence of increasing amount of GST- K85A-EhAK1. EhCaBP1 was then immunoprecipitated with anti-EhCaBP1 antibody and the blot was developed EhAK1.
(TIF)

Figure S9 **Effect of down regulation of EhCaBP1 during phagocytosis.** *E. histolytica* cells expressing anti sense EhCaBP1 RNA were incubated with RBC for indicated time interval (3, 5 and 10 min) at 37°C. The cells were then fixed and immunostained with TRITC-phalloidin. Graph shows quantitative analysis of number of phagocytic cups formed in these cell lines.
(TIF)

Figure S10 **Complete b/y series of phospho-peptide of Ehactin.** Table shown complete ions series of phospho-peptide of Ehactin.
(TIF)

Figure S11 **Secondary structure alignment of** ***E. histolytica*** **actin with other actins.** Sequence alignment of actin from *E. histolytica* (B1N2P0), *O. Cuniculus* (PDB-1IJJ), *D. discoideum* (PDB-1NLV) and *H. Sapiens* (PDB-3BYH) with superimposed secondary structure. The T107 site, which is conserved and is present in beta sheet region is boxed. Alignment figure produced with ESPript.
(TIF)

Figure S12 **3D modelling of wild type Ehactin and mutant T107A Ehactin.** (A) 3-D structure of Ehactin was modelled using actin of *D. dicoideum* (PDB 3Ci5.1.A) as template (which had sequence identity of 90.8%, GMQE 0.99 and QMEAN4-0.59 with Ehactin) using online SWISS-MODEL software. The superimposed structure of Ehactin and rabbit skeletal muscle actin (PDB 1iJJ) was generated using online FATCAT software which was further analyzed on PYMOL. RMSD value of superimposed Ehactin and Rabbit skeletal muscle actin was 2.12. (B) 3-D structure of Ehactin and mutant T107A Ehactin was modelled and superimposed same as described in A) which had RMSD value of 0.001.
(TIF)

Figure S13 **Effect of EhCaBP1 on kinase activity of EhAK1.** Effect of EhCaBP1 on EhAK1 activity. Rabbit actin (2µg) was incubated with EhAK1 (2µg) and mutant K85A-EhAK1 (2µg) in kinase buffer as described in “Materials and Methods”.
(TIF)

Figure S14 **Specificity of phospho-specific Ehactin antibody and** ***in vivo*** **distribution of p-Ehactin during phagocytosis.** A. Western blot analysis of immunoprecipitate phosphorylated GFP-Ehactin, dephosphorylated GFP-Ehactin and GFP-T107A mutant using anti-pEhactin antibody at dilution 1∶100. B. *E. histolytica* cells were incubated with RBC for 3 min at 37°C. The cells were then fixed and immunostained with anti-pEhactin. F-actin was stained with TRITC-phalloidin. Arrow heads indicate phagocytic cups. Bar represents 5µm.
(TIF)

Figure S15 **Western blot analysis of phospho-rabbit actin.** Purified non-phosphorylated and *in vitro* phosphorylated rabbit skeletal muscle actin by EhAK1 were separated on a SDS-PAGE and transferred onto a PVDF membrane. The phosphorylated rabbit actin was visualized by immunostaining using anti-p-T107 anti body at dilution 1: 100.
(TIF)

Figure S16 **Effect of kinase inhibitors on p-Ehactin levels.** Cells were treated with kinase inhibitors genistien (200µM) and staurosporine (2µM) for 30 min. Cell lysate were prepared and were subjected to immunoprecipitation with anti-Ehactin or anti-EhC2PK antibodies (as control) followed by western blots.
(TIF)

Figure S17 ***In vivo*** **over-expression of GFP-tagged wild type and T107A actin.** Western blot analysis of cell lines expressing either wild type GFP-Ehactin or mutant GFP-T107A actin with anti-Ehactin or anti-GFP antibody as indicated.
(TIF)

Movie S1 **Live cell imaging of amoebic cells expressing GFP-EhAK1.** The movie represents temporal changes in amoeba expressing GFP-EhAK1 during erythrophagocytosis. The enrichment of EhAK1 takes place within 9 s at the site of RBC attachment to the cell surface of amoeba. Bar represents 10 µm.
(AVI)

Movie S2 **Live cell imaging of normal amoebic cells phagocytosing labelled RBCs.**
(AVI)

Movie S3 **Live cell imaging of K85A-EhAK1 over-expressing cells phagocytosing labelled RBCs.**
(AVI)

Table S1 **List of some peptide identified by LC/MS as a EhAK1 binding proteins.**
(DOCX)
