# Supplementary figures and images for: A Novel Alpha Kinase EhAK1 Phosphorylates Actin and Regulates Phagocytosis in Entamoeba histolytica
Source: PLoS Pathog. 2014 Oct 9;10(10):e1004411. doi: 10.1371/journal.ppat.1004411 (PMC4192601; doi:10.1371/journal.ppat.1004411)

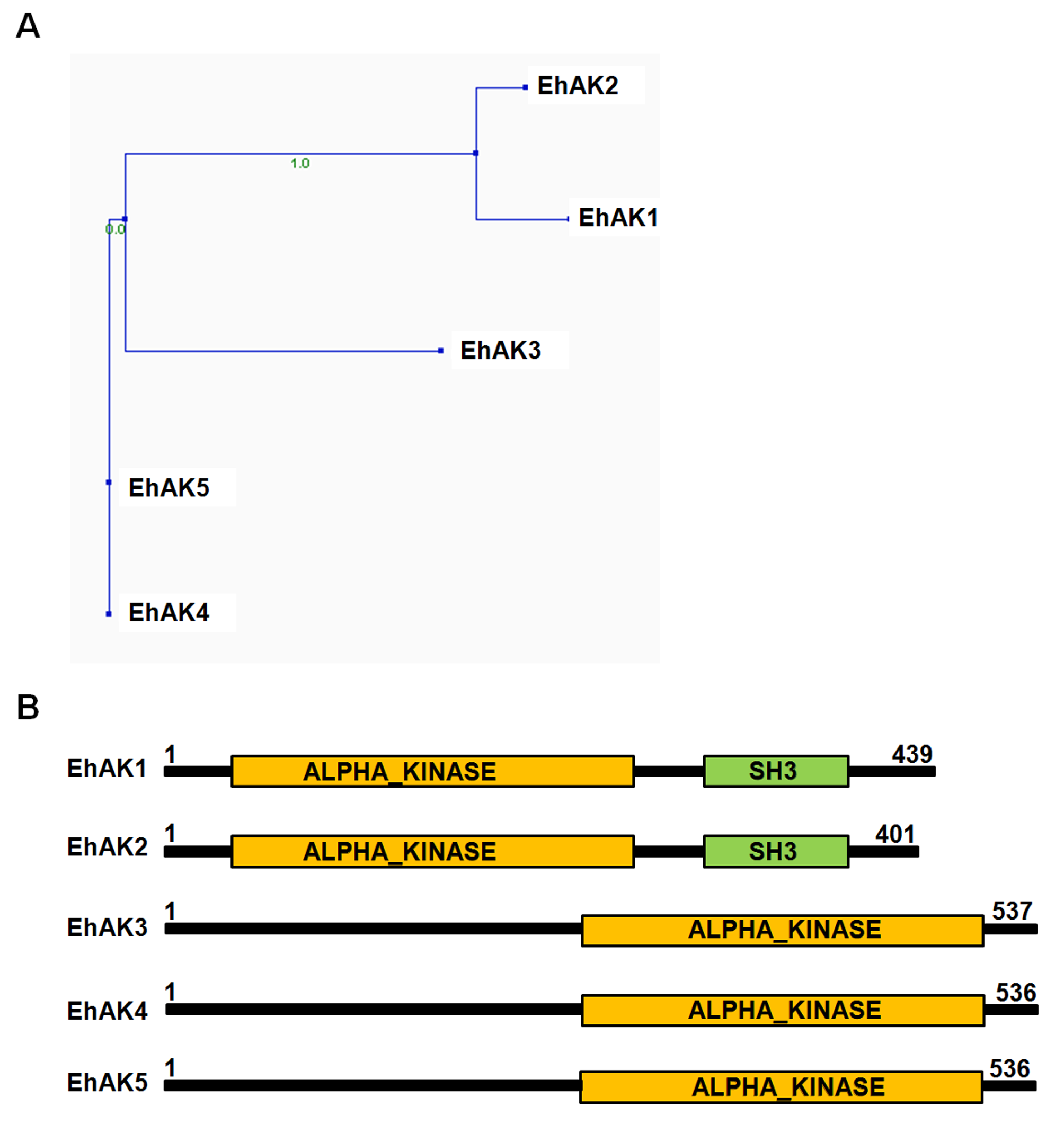

Supplement: Figure S1 — Domain organization and phylogenetic analysis of different alpha kinases encoded by E. histolytica . (A) Phylogenetic analysis of the alpha kinase family of E. histolytica. A PSI-Blast search was done to identify all sequenced alpha kinases from genome database using EhAK1 alpha kinase domain. For some of the predicted sequences that did not display alpha kinase domain using Scan prosite, reciprocal PSI-Blast searches were performed to ensure that they were indeed homologs of alpha kinases. Selected sequences were then aligned using ClustalW2. A phylogenetic tree was generated using PHYML. Bootstrap values of the major branches are indicated. (B) (A) Schematic presentation of domain organization of different alpha kinases EhAK1 (XP_656642), EhAK2 (XP_651695), EhAK3 (XP_652177), EhAK4 (XP_654603), and EhAK5 (XP_654482) encoded by E. histolytica genome. (TIF) [file ppat.1004411.s001.tif]

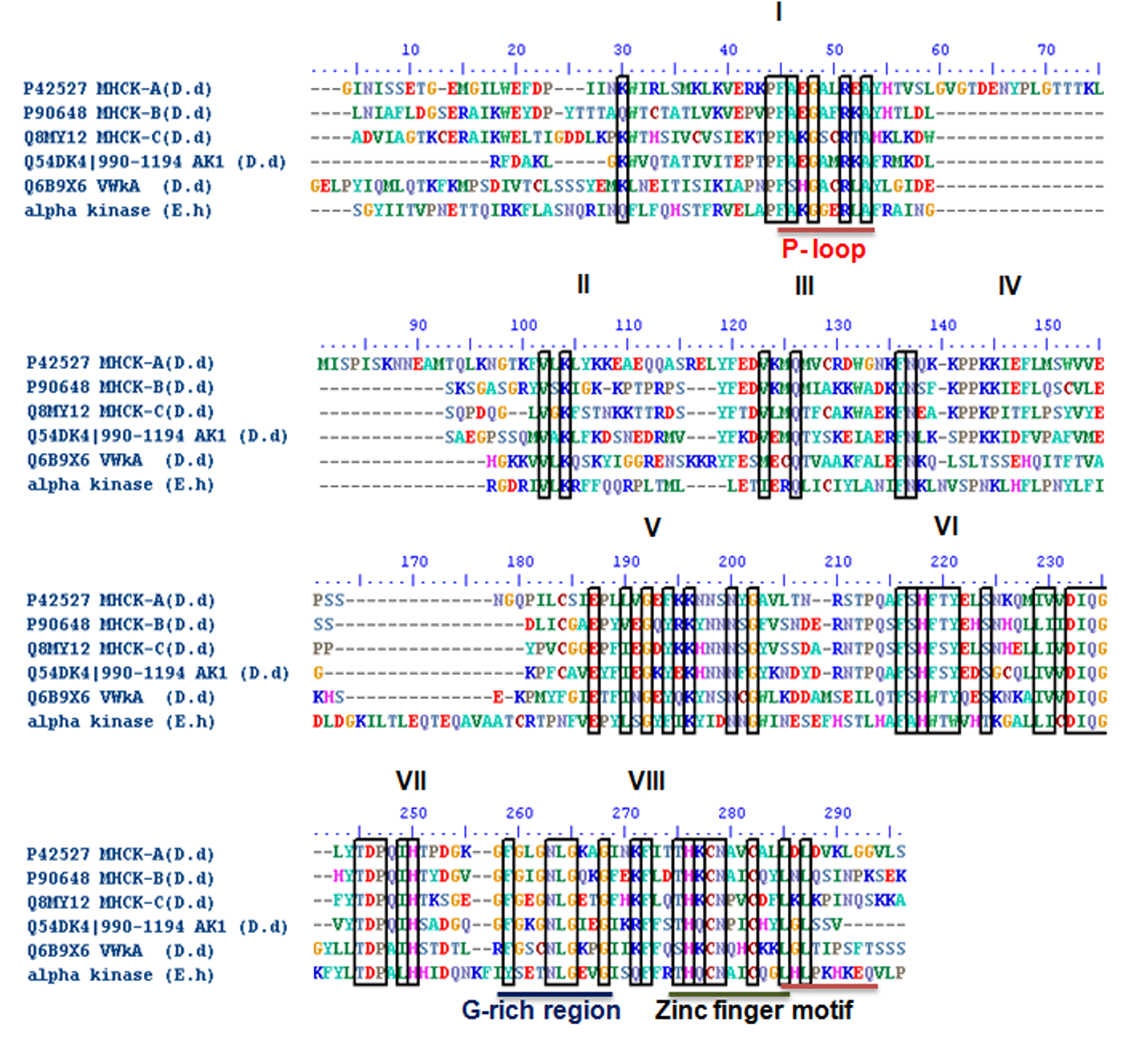

Supplement: Figure S2 — Multiple sequence alignment of the kinase domain of EhAK1 with respective domains of alpha kinases from D. discoideum. D. discoideum alpha-kinases (MHCK A-P42527, MHCKB-P90648, MHCKC-Q8MY12, AK1-Q54DK4, VWkA-Q6B9X6) and EhAK1 alpha-kinase domain (C4M9G9) were aligned using ClustalW2 and clustered into eight sub domains. D. discoideum (D.d), E. histolytica (E.h) and sequences representing P-loop, G-rich region and Zinc finger motif are indicated. Invariant K85 that is predicted to be involved in nucleoside binding site is also shown. (TIF) [file ppat.1004411.s002.tif]

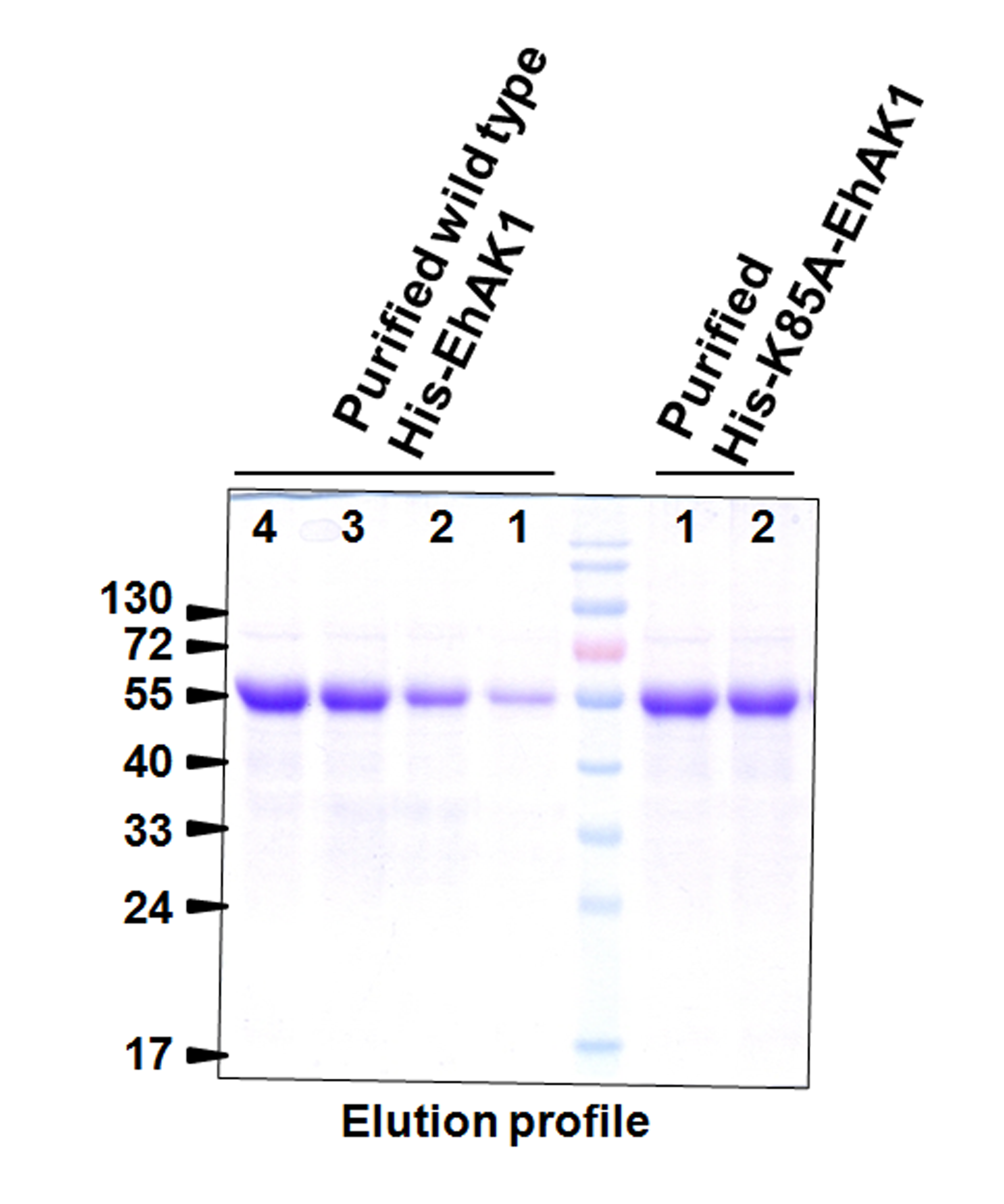

Supplement: Figure S3 — Purification of recombinant wild type EhAK1 and K85A-EhAK1. SDS page analysis of purified HIS-tagged wild type EhAK1 and mutant K85A-EhAK1 is shown. (TIF) [file ppat.1004411.s003.tif]

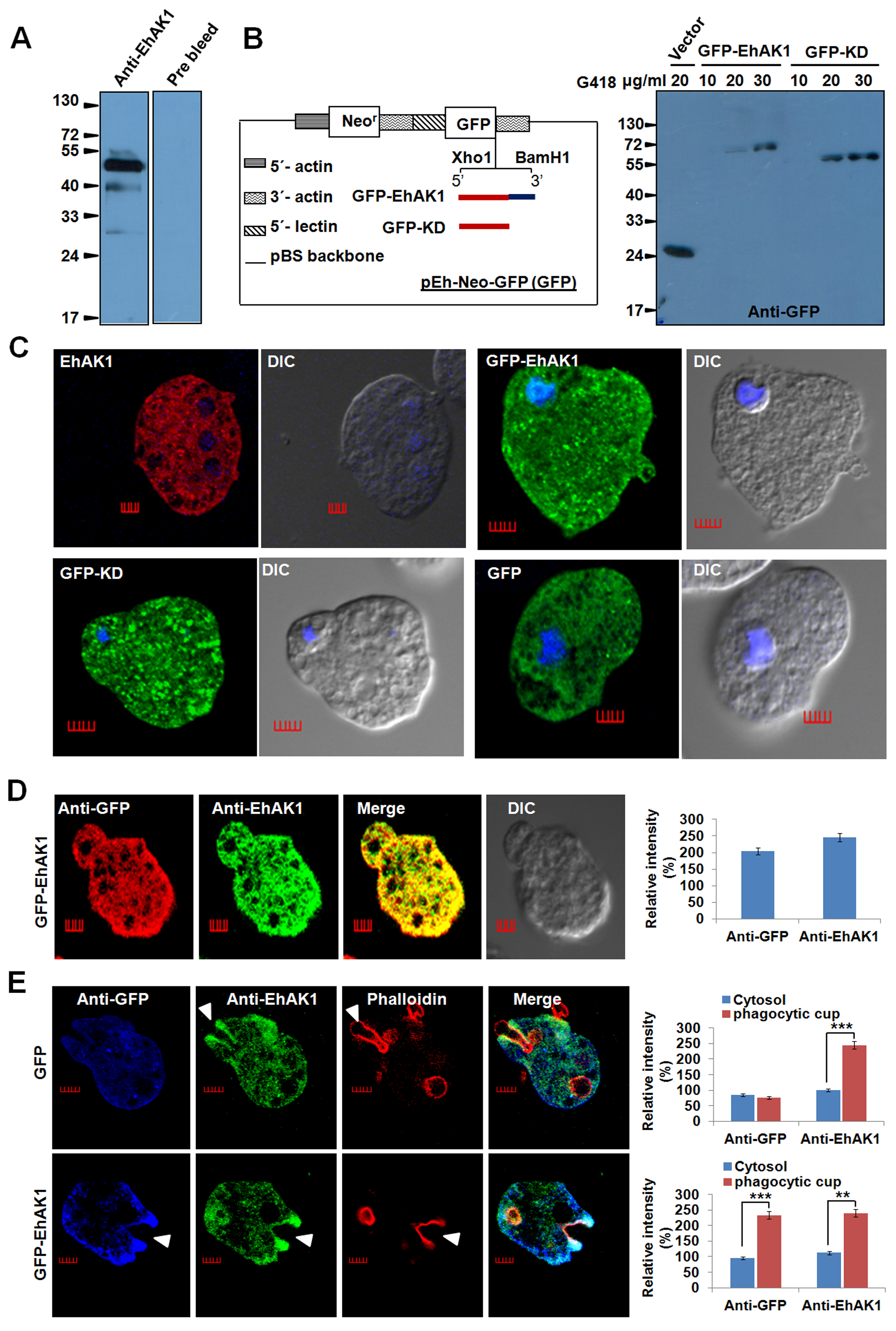

Supplement: Figure S4 — In vivo expression of EhAK1. (A) Western blot analysis was used for checking the specificity of raised Anti-EhAK1 antibody in Entamoeba lysate (Anti-EhAK1 1;1000). Pre-bleed was taken as control. (B) Schematic representation of the constitutive expression system used for expression of GFP-conjugated proteins in amoebic cells. Western blot analysis for detection of endogenous EhAK1, overexpressed GFP, GFP-EhAK1 and GFP-KD. Total cell lysate (50 µg) was separated in SDS-PAGE and were transferred on to a PVDF membrane for immunodetection. Anti-GFP antibody was used at 1∶3000 dilution. The bound antibodies were identified by an appropriate peroxidase-labelled secondary antibody raised against rabbit immunoglobulins and visualized with ECL reagents. (C) and (D) Immunolocalization of EhAK1 in indicated E. histolytica cells. Transfectants containing GFP-EhAK1, GFP-KD and only GFP vector or normal amoebic cells were grown in presence or absence of 30µg/ml G418. Immunofluorescence was performed using anti-EhAK1 and anti-GFP antibodies followed by Alexa-555 (red) and Alexa-488 (green) or Pacific blue-410 respectively. Nucleus was stained using Hoechst (Blue). (Scale bar, 5 µm; DIC, differential interference contrast). Quantitative analysis of fluorescent signals was done as in Fig. 1d. (E) Imaging of EhAK1 during erythrophagocytosis in cells containing GFP or GFP-EhAK1 constructs. Cells were grown for 48 h and incubated with RBC for 5 min at 37°C. Immunostaining was performed using anti-GFP or anti-EhAK1 antibodies followed by Pacific blue-410 and Alexa-488 respectively. F-actin was stained with TRITC-phalloidin. Arrowheads indicate phagocytic cups. Quantitative analysis of fluorescent signals was done as in Fig. 1d. Bar represents 5 µm. (TIF) [file ppat.1004411.s004.tif]

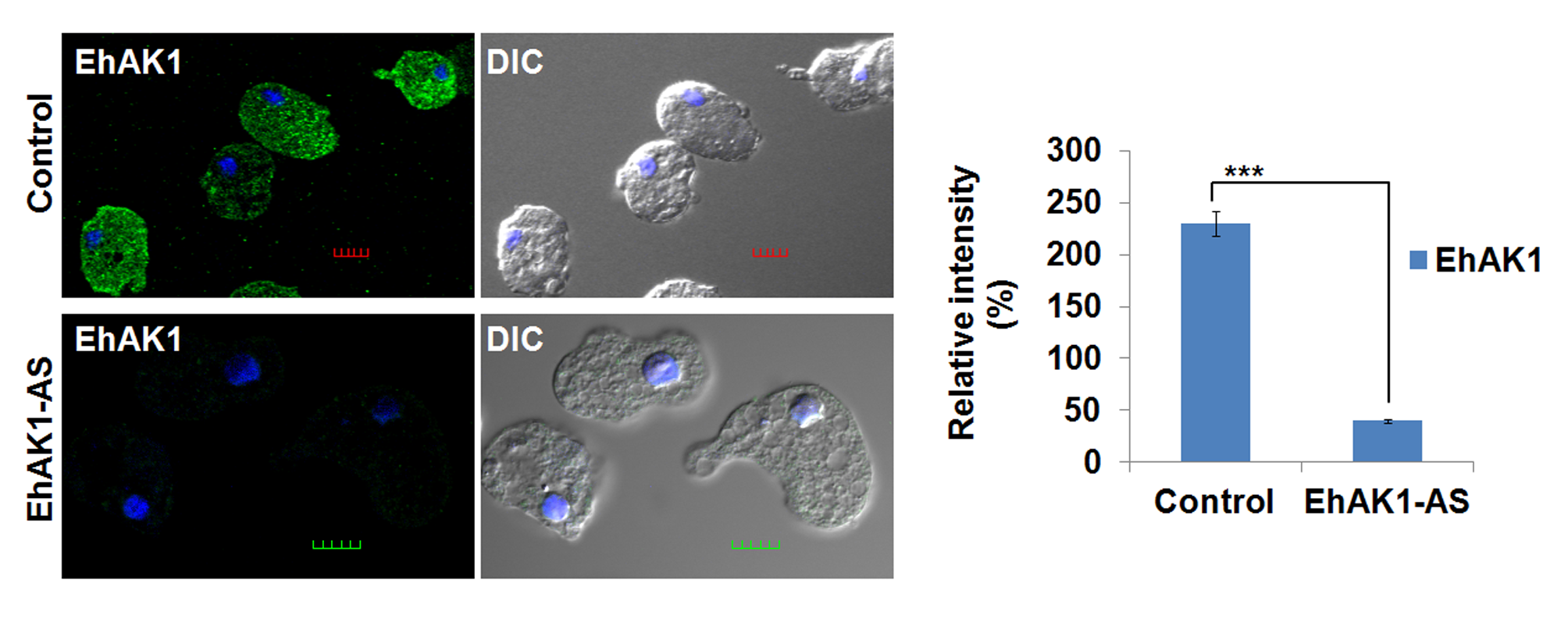

Supplement: Figure S5 — Over-expression of antisense RNA of EhAK1. Imaging of EhAK1 in E. histolytica cells. Normal amoeba and EhAK1-AS were grown for 48 h in presence or absence of 20 µg/ml tet. Immunostaining was performed using anti-EhAK1 antibody followed by Alexa-488. F-actin was stained with TRITC-phalloidin. Quantitative analysis of fluorescent signals obtained by immunostaining of EhAK1 from different locations in E. histolytica cells and EhAK1 transfectants. For analysis, five random regions were selected and average intensity was computed for each region. This was repeated for five such cells (N = 5, bars represent standard error). Bar represents 10 µm. (TIF) [file ppat.1004411.s005.tif]

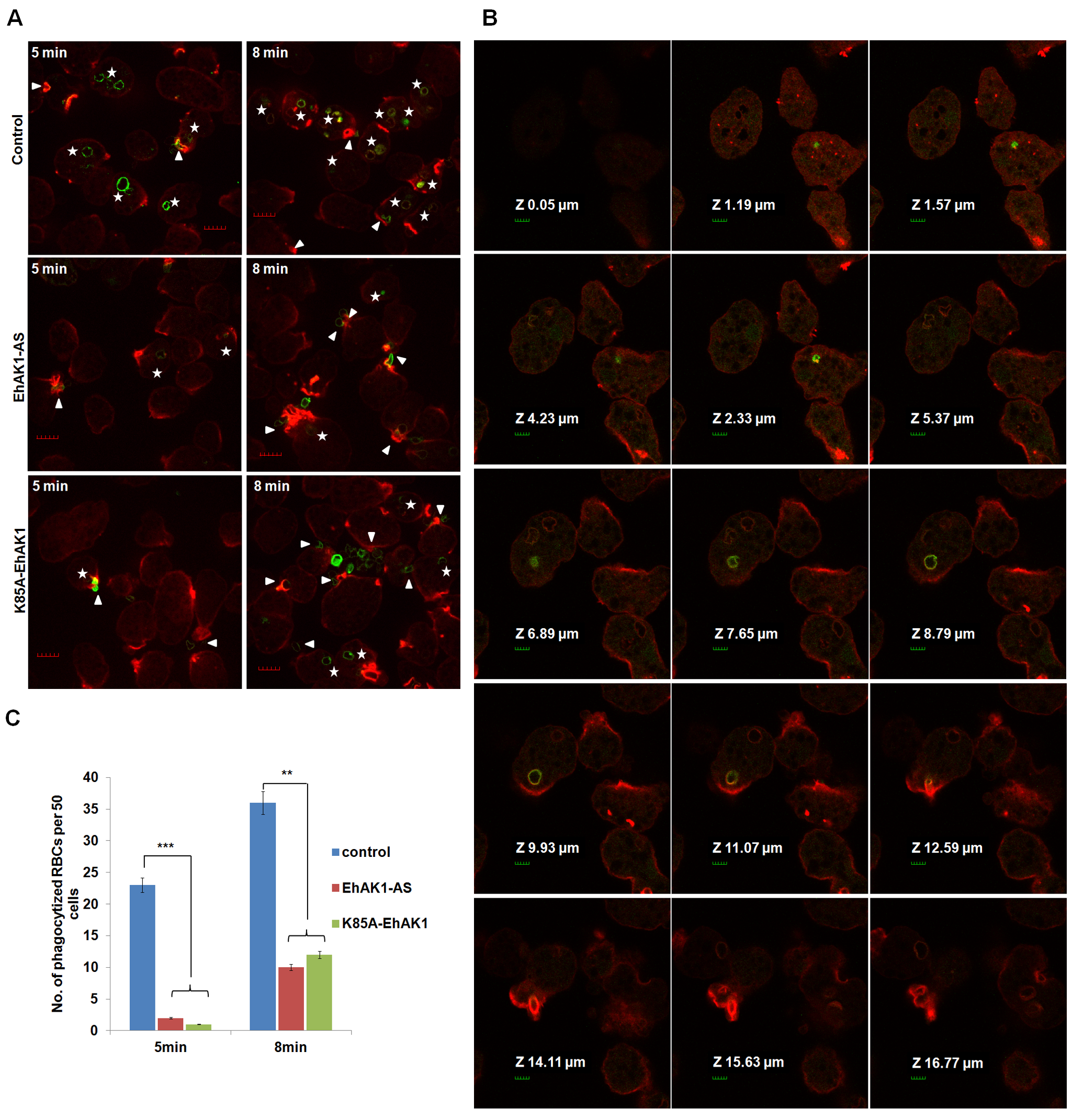

Supplement: Figure S6 — Phagocytic uptake of fluorescent labelled RBCs. (A) Amoebic cells with and without indicated constructs were incubated with fluorescent labelled RBCs for indicated time at 37°C. These cells were then fixed and stained with TRITC-Phalloidin. Arrows show attached RBCs at the site of phagocytosis and star marks the phagocytized RBCs. (B) Z-section of the amoebic cells incubated with fluorescent RBC's (Green) and stained with TRITC-phalloidin (Red). (C) Quantitative analysis was carried out by selecting randomly fifty cells from each experiment and the numbers of phagocytized RBCs present in all cells were counted. (TIF) [file ppat.1004411.s006.tif]

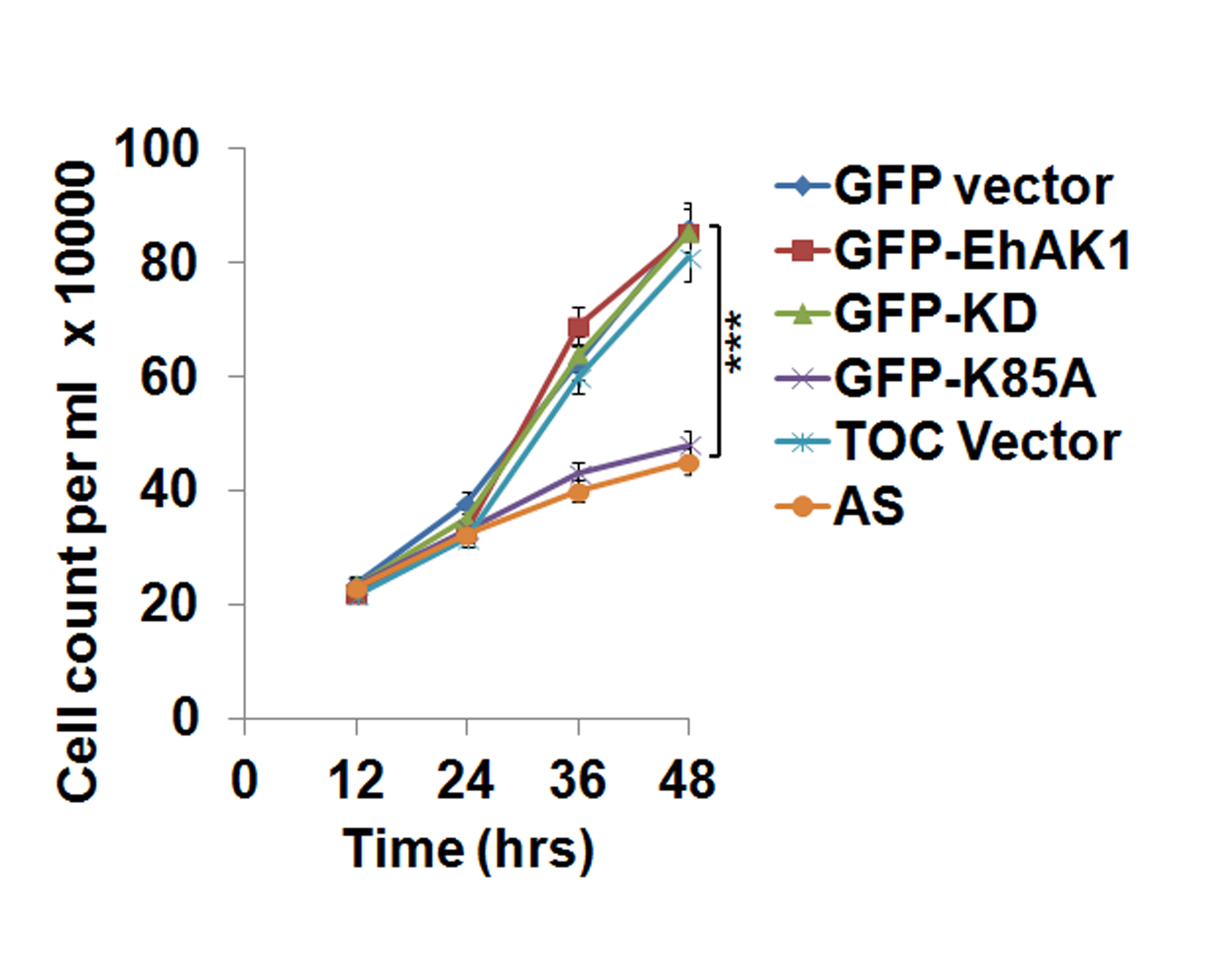

Supplement: Figure S7 — Proliferation of E. histolytica cells in presence of different constructs. All cells were grown in presence of 20 µg/ml hygromycin/G418 and tetracycline was added to the medium at 20 µg/ml at 0 h. Cells were grown in 5 ml culture tubes in triplicate for all the experiments and counting was carried out using a haemocytometer, after chilling the tube for 5 min. (TIF) [file ppat.1004411.s007.tif]

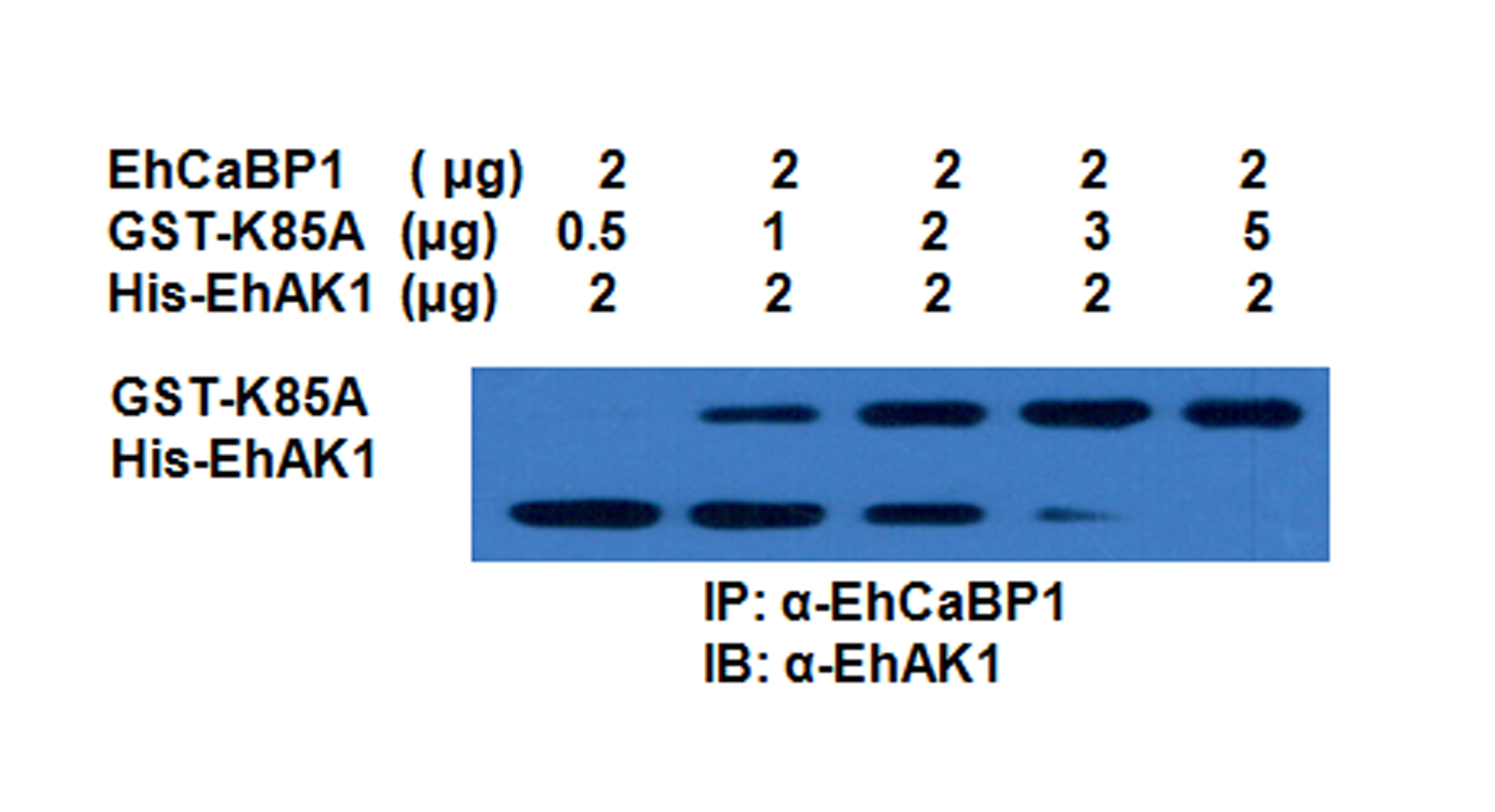

Supplement: Figure S8 — In vitro competition assay. In vitro competition assay of K85A-EhAK1 mutant and wild type EhAK1. Recombinant wild type EhAK1 (2µg) was incubated with EhCaBP1 (2 µg) in presence of increasing amount of GST- K85A-EhAK1. EhCaBP1 was then immunoprecipitated with anti-EhCaBP1 antibody and the blot was developed EhAK1. (TIF) [file ppat.1004411.s008.tif]

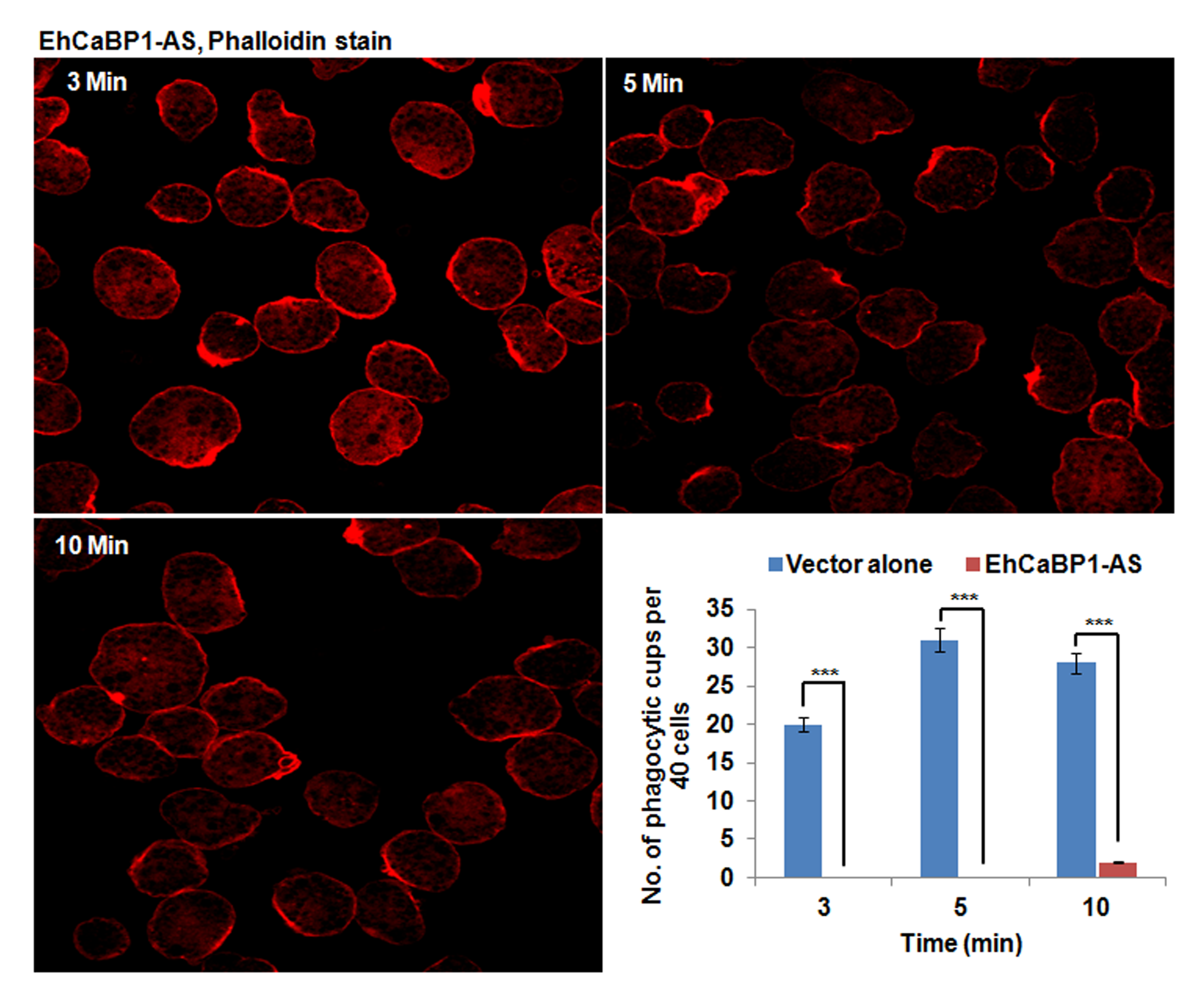

Supplement: Figure S9 — Effect of down regulation of EhCaBP1 during phagocytosis. E. histolytica cells expressing anti sense EhCaBP1 RNA were incubated with RBC for indicated time interval (3, 5 and 10 min) at 37°C. The cells were then fixed and immunostained with TRITC-phalloidin. Graph shows quantitative analysis of number of phagocytic cups formed in these cell lines. (TIF) [file ppat.1004411.s009.tif]

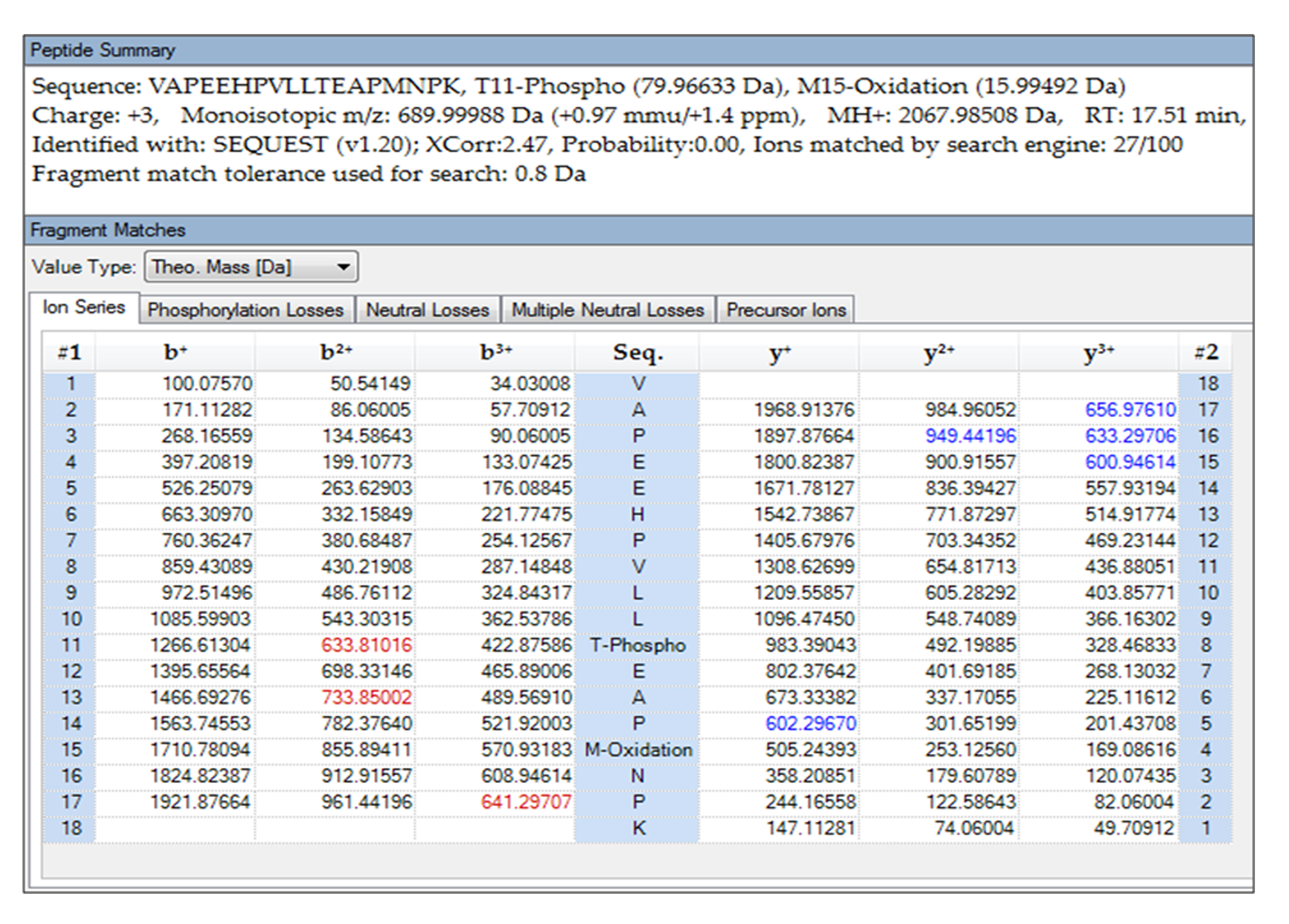

Supplement: Figure S10 — Complete b/y series of phospho-peptide of Ehactin. Table shown complete ions series of phospho-peptide of Ehactin. (TIF) [file ppat.1004411.s010.tif]

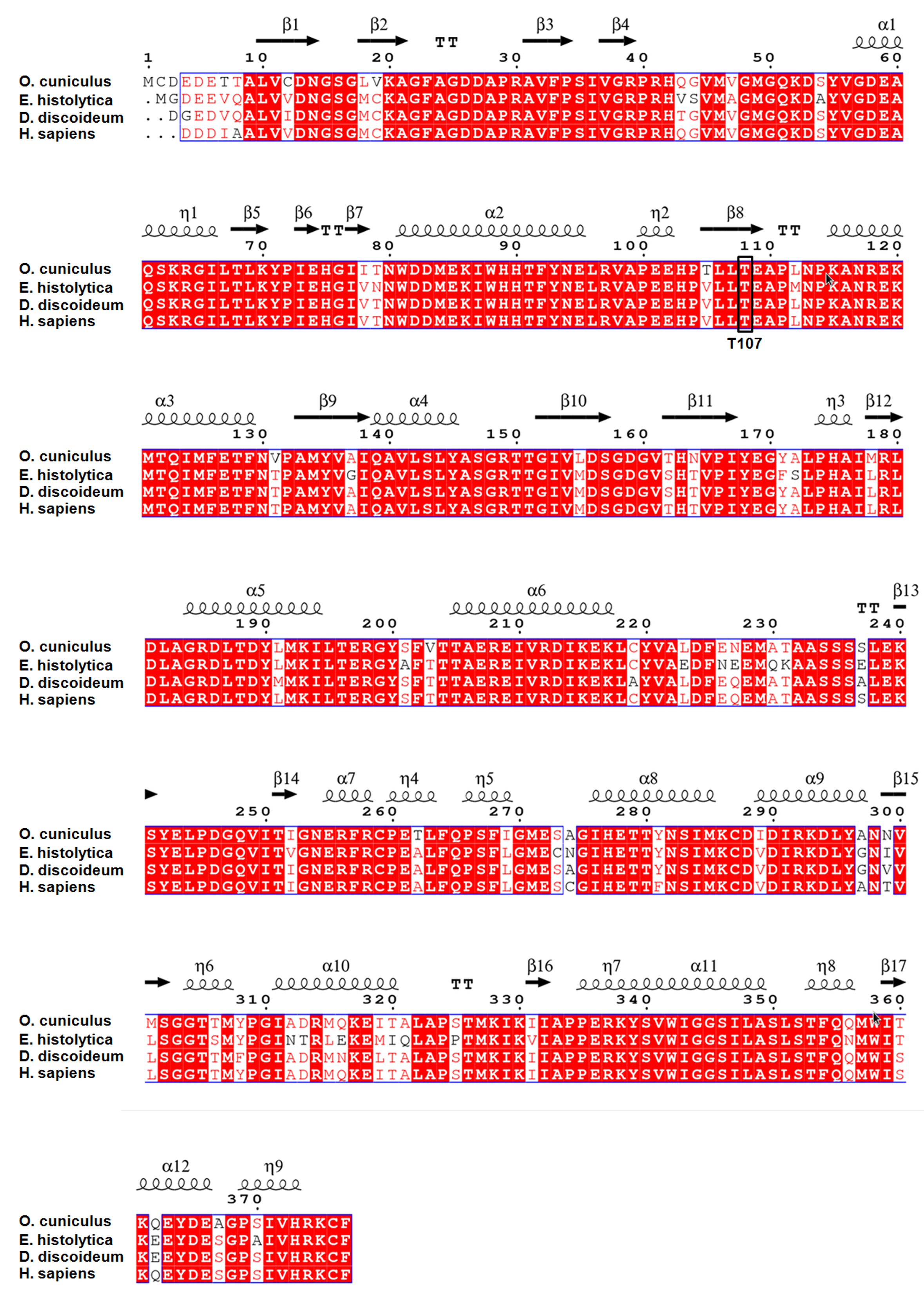

Supplement: Figure S11 — Secondary structure alignment of E. histolytica actin with other actins. Sequence alignment of actin from E. histolytica (B1N2P0), O. Cuniculus (PDB-1IJJ), D. discoideum (PDB-1NLV) and H. Sapiens (PDB-3BYH) with superimposed secondary structure. The T107 site, which is conserved and is present in beta sheet region is boxed. Alignment figure produced with ESPript. (TIF) [file ppat.1004411.s011.tif]

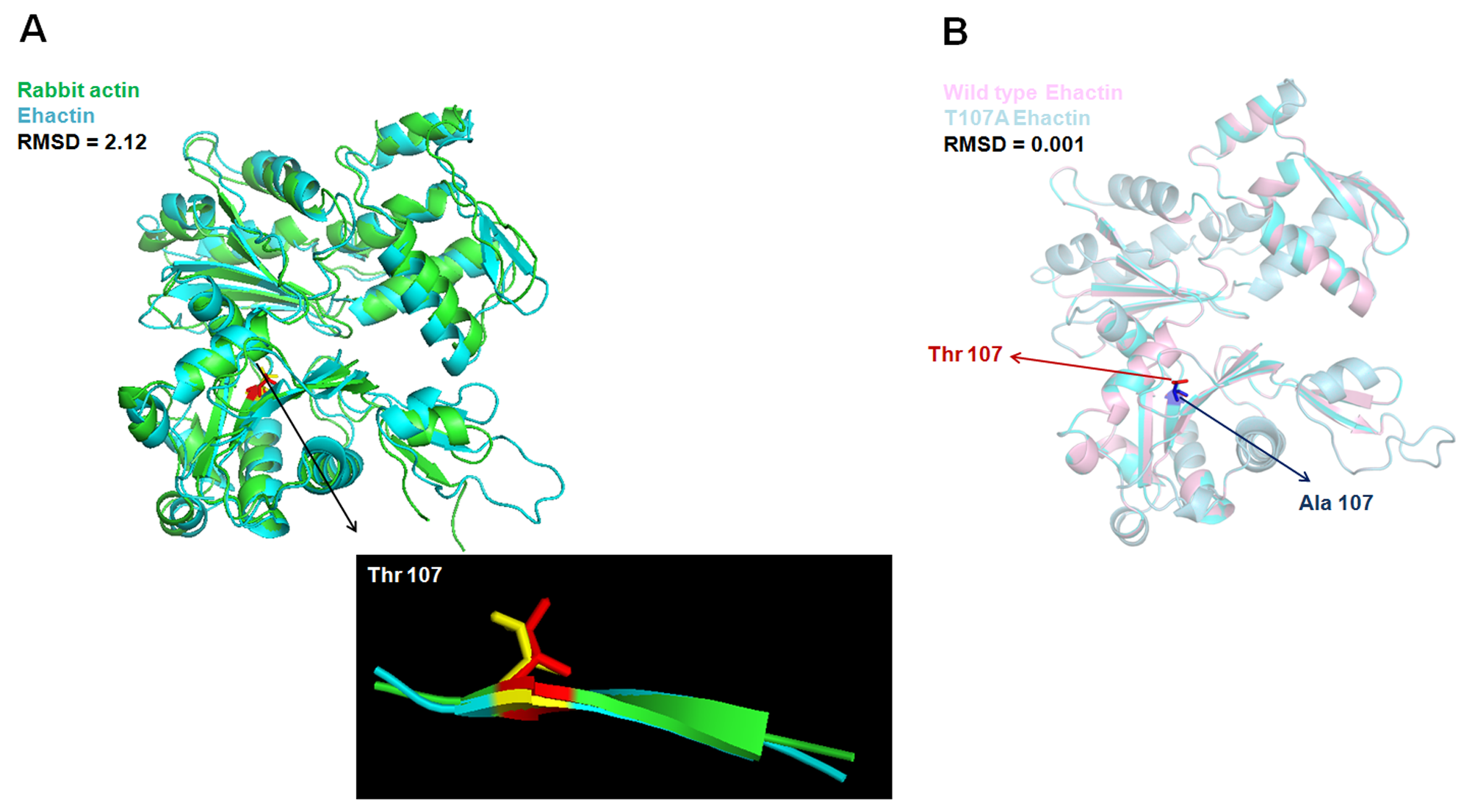

Supplement: Figure S12 — 3D modelling of wild type Ehactin and mutant T107A Ehactin. (A) 3-D structure of Ehactin was modelled using actin of D. dicoideum (PDB 3Ci5.1.A) as template (which had sequence identity of 90.8%, GMQE 0.99 and QMEAN4-0.59 with Ehactin) using online SWISS-MODEL software. The superimposed structure of Ehactin and rabbit skeletal muscle actin (PDB 1iJJ) was generated using online FATCAT software which was further analyzed on PYMOL. RMSD value of superimposed Ehactin and Rabbit skeletal muscle actin was 2.12. (B) 3-D structure of Ehactin and mutant T107A Ehactin was modelled and superimposed same as described in A) which had RMSD value of 0.001. (TIF) [file ppat.1004411.s012.tif]

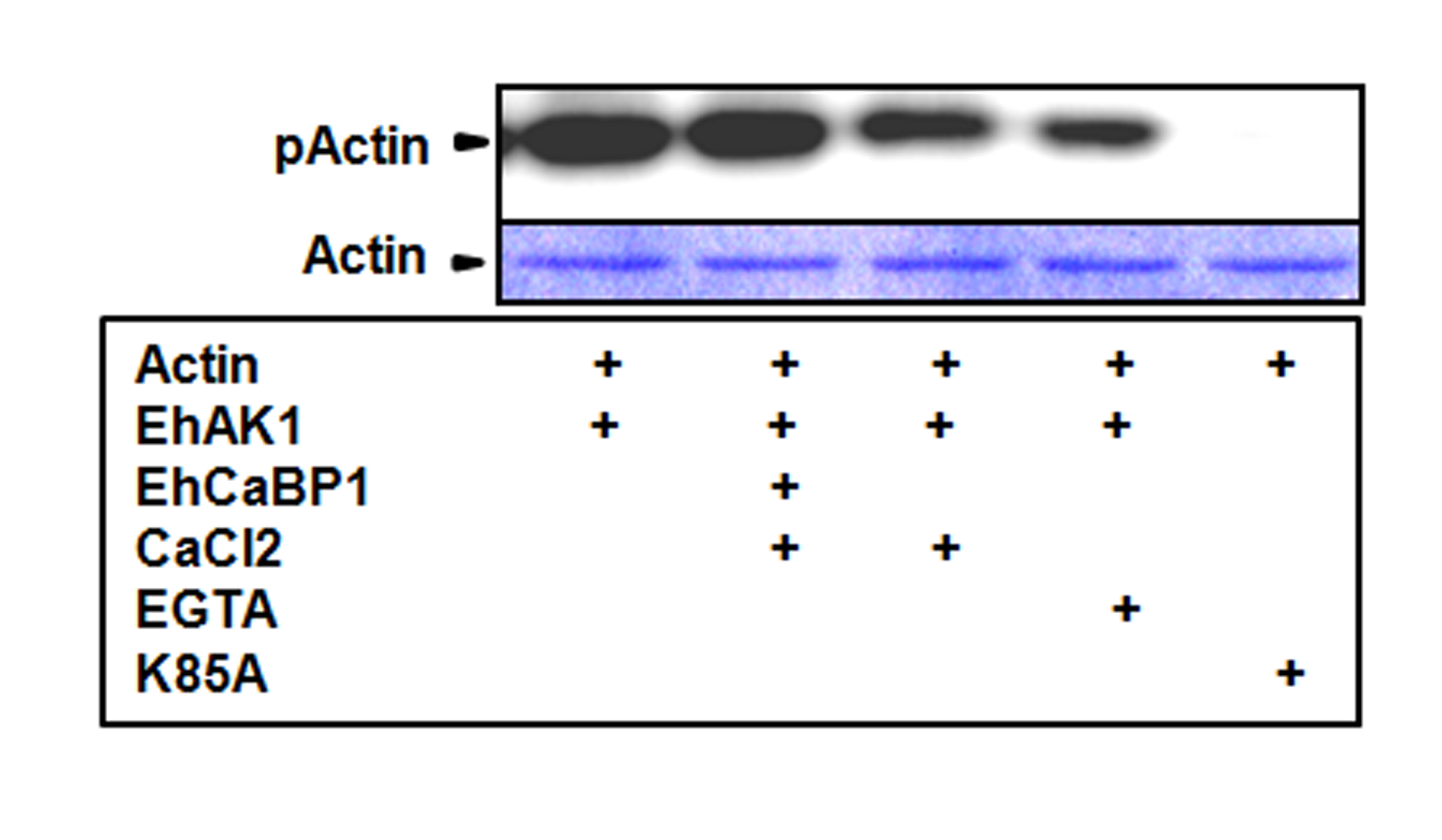

Supplement: Figure S13 — Effect of EhCaBP1 on kinase activity of EhAK1. Effect of EhCaBP1 on EhAK1 activity. Rabbit actin (2µg) was incubated with EhAK1 (2µg) and mutant K85A-EhAK1 (2µg) in kinase buffer as described in “Materials and Methods”. (TIF) [file ppat.1004411.s013.tif]

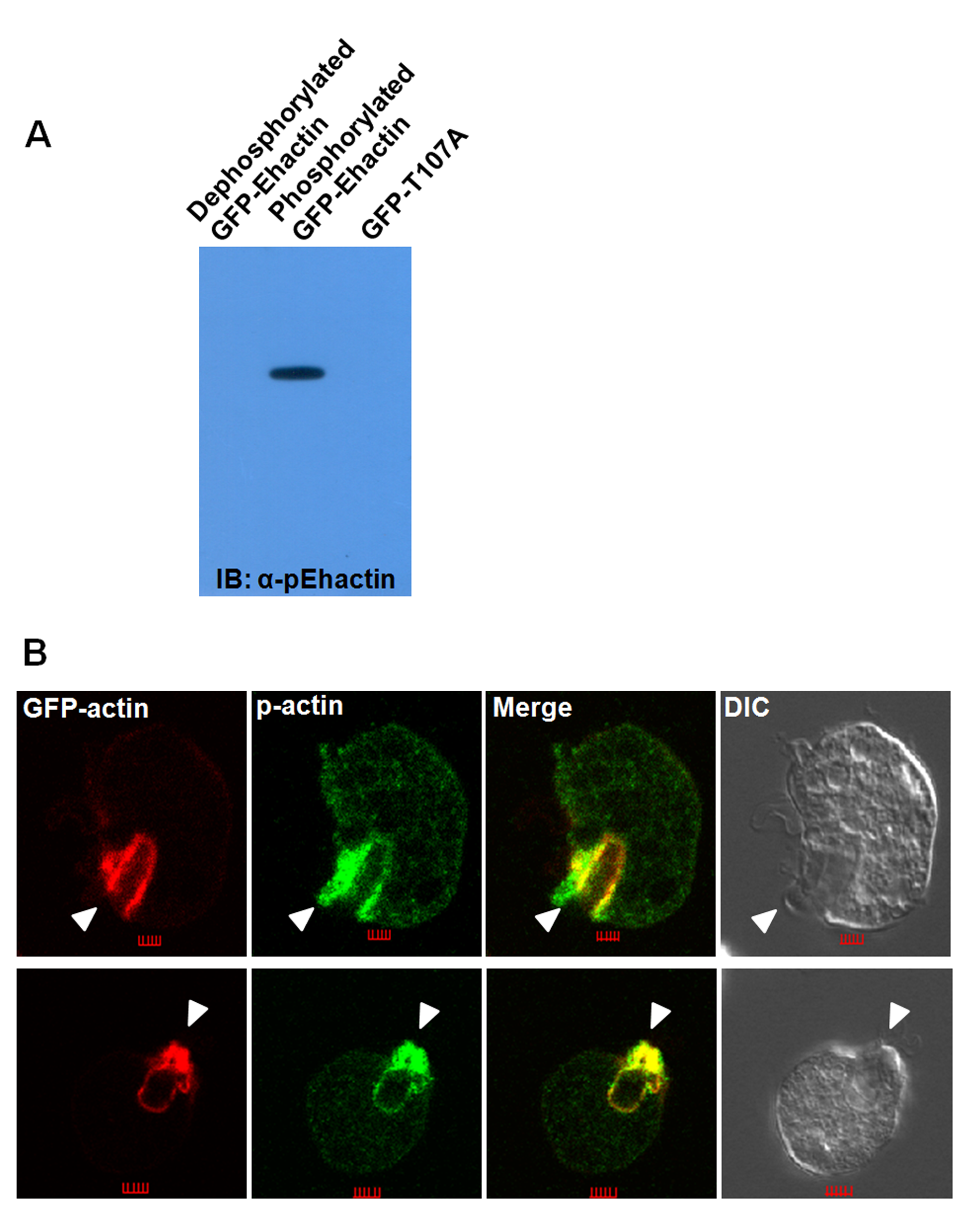

Supplement: Figure S14 — Specificity of phospho-specific Ehactin antibody and in vivo distribution of p-Ehactin during phagocytosis. A. Western blot analysis of immunoprecipitate phosphorylated GFP-Ehactin, dephosphorylated GFP-Ehactin and GFP-T107A mutant using anti-pEhactin antibody at dilution 1∶100. B. E. histolytica cells were incubated with RBC for 3 min at 37°C. The cells were then fixed and immunostained with anti-pEhactin. F-actin was stained with TRITC-phalloidin. Arrow heads indicate phagocytic cups. Bar represents 5µm. (TIF) [file ppat.1004411.s014.tif]

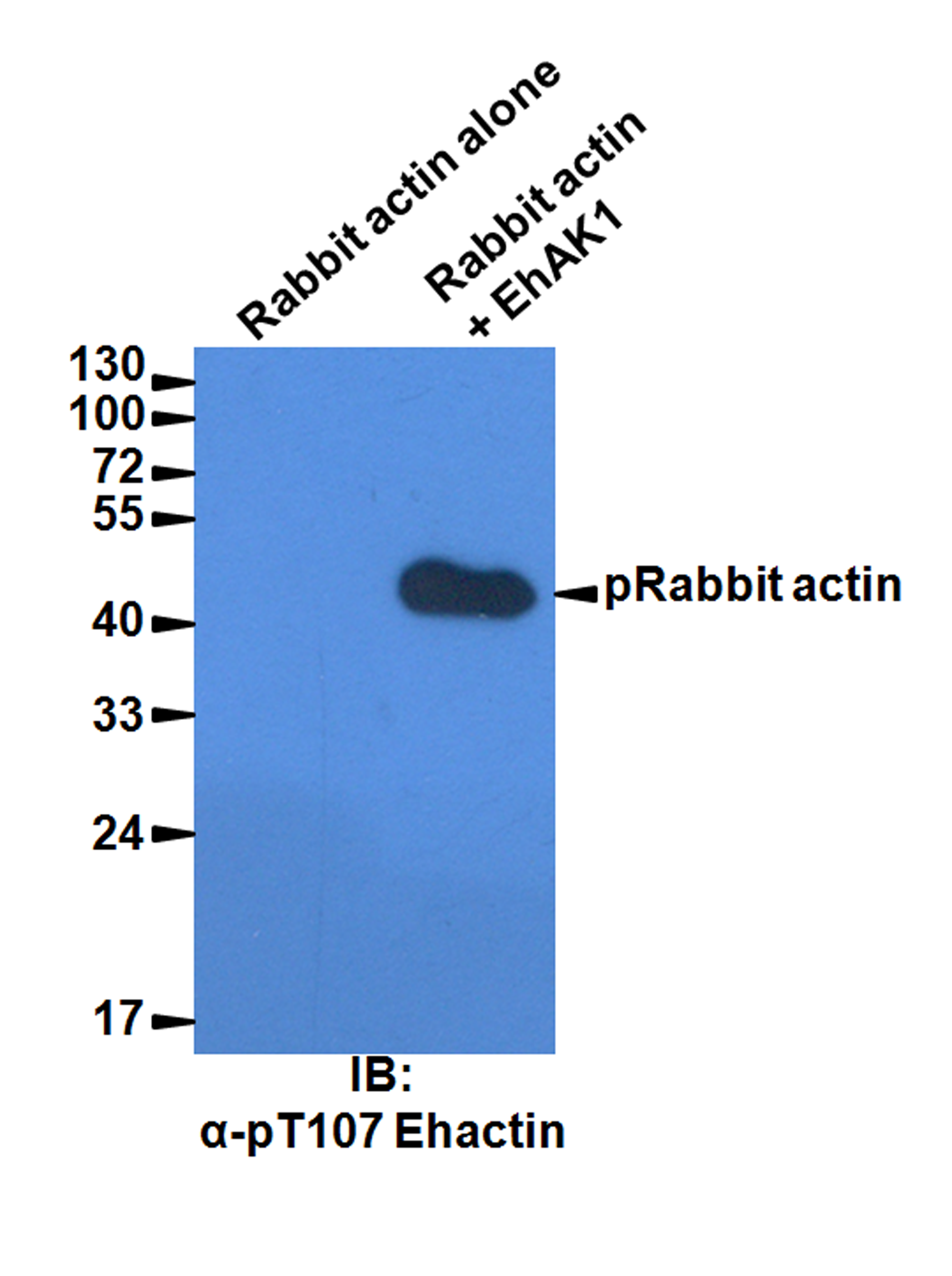

Supplement: Figure S15 — Western blot analysis of phospho-rabbit actin. Purified non-phosphorylated and in vitro phosphorylated rabbit skeletal muscle actin by EhAK1 were separated on a SDS-PAGE and transferred onto a PVDF membrane. The phosphorylated rabbit actin was visualized by immunostaining using anti-p-T107 anti body at dilution 1: 100. (TIF) [file ppat.1004411.s015.tif]

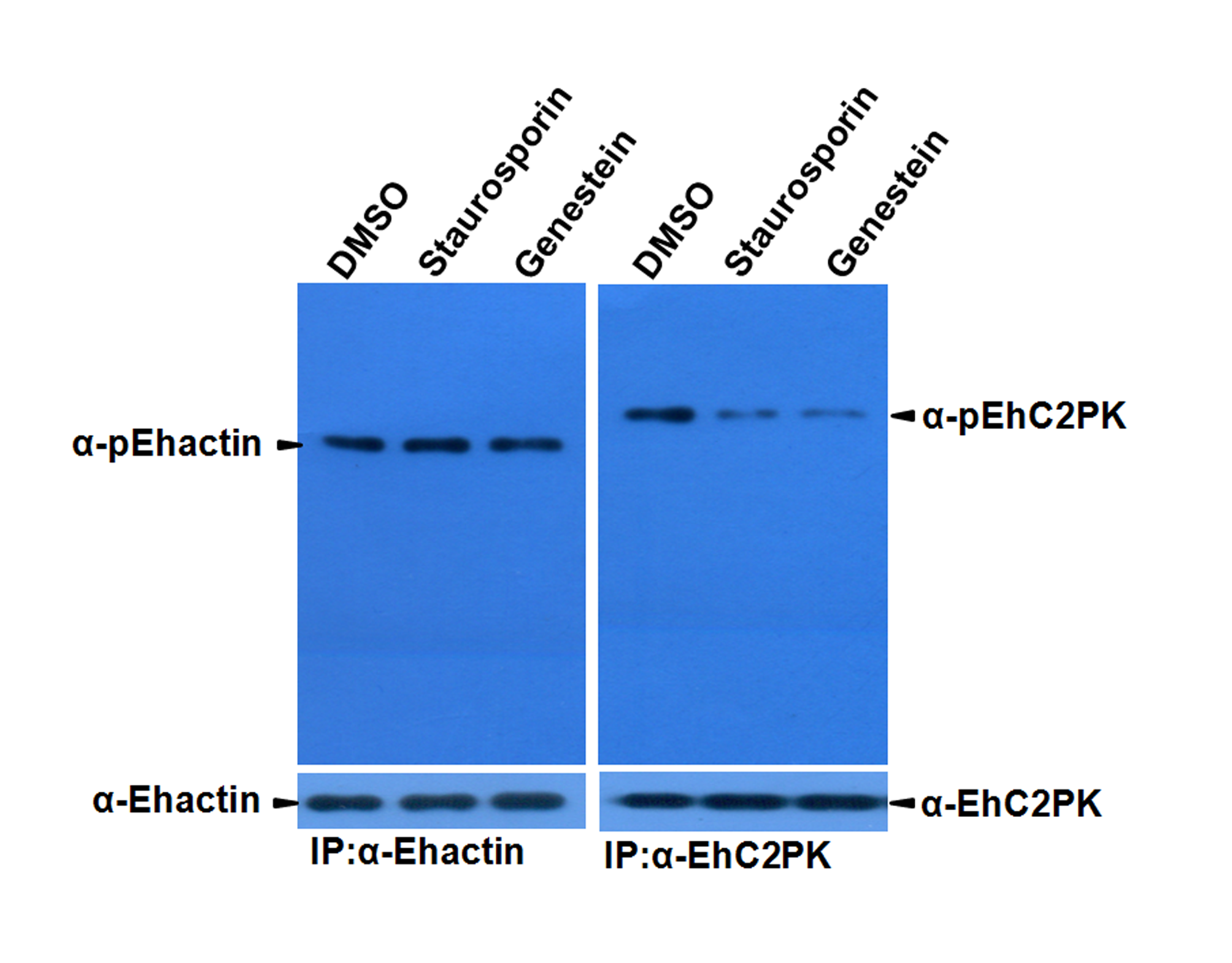

Supplement: Figure S16 — Effect of kinase inhibitors on p-Ehactin levels. Cells were treated with kinase inhibitors genistien (200µM) and staurosporine (2µM) for 30 min. Cell lysate were prepared and were subjected to immunoprecipitation with anti-Ehactin or anti-EhC2PK antibodies (as control) followed by western blots. (TIF) [file ppat.1004411.s016.tif]

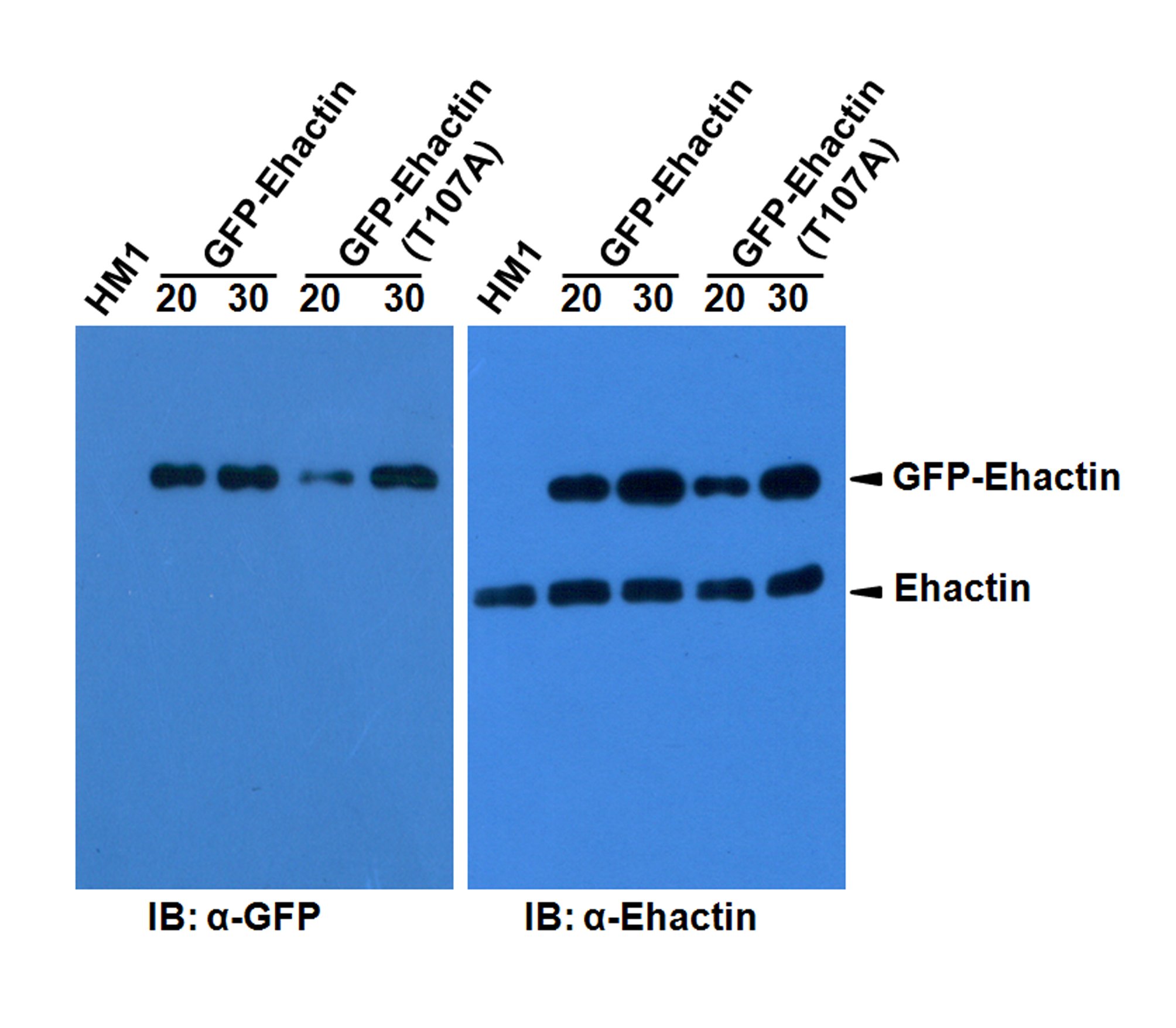

Supplement: Figure S17 — In vivo over-expression of GFP-tagged wild type and T107A actin. Western blot analysis of cell lines expressing either wild type GFP-Ehactin or mutant GFP-T107A actin with anti-Ehactin or anti-GFP antibody as indicated. (TIF) [file ppat.1004411.s017.tif]
