# Supplementary material for: A Novel Alpha Kinase EhAK1 Phosphorylates Actin and Regulates Phagocytosis in Entamoeba histolytica
Source: PLoS Pathog. 2014 Oct 9;10(10):e1004411. doi: 10.1371/journal.ppat.1004411 (PMC4192601; doi:10.1371/journal.ppat.1004411)
Supplement: Table S1 — List of some peptide identified by LC/MS as a EhAK1 binding proteins. (DOCX) [file ppat.1004411.s021.docx]

**Table S1. List of some peptide identified by LC/MS as a EhAK1 binding proteins.**

| **Accession** | **Description** | **Coverage** | **Score** | **MW**  **(kDa)** |
| --- | --- | --- | --- | --- |
| C4MAC9_ENTHI | Elongation factor 1-alpha EHI_052400 GTP binding; GTPase activity; cytoplasm; translation elongation factor activity | 46.38% | 94.99 | 48.4 |
| B1N2P0_ENTHI | Actin EHI_107290 ATP binding; cytoplasm; cytoskeleton | 45.74% | 187.74 | 42 |
| C4M0F4_ENTHI | 14-3-3 protein 3 EHI_006810 protein domain specific binding | 40.83% | 59.14 | 27.5 |
| C4M9G9_ENTHI | Protein kinase, putative EHI_105830 ATP binding; protein phosphorylation; protein serine/threonine kinase activity | 30.07% | 97.39 | 49.9 |
| C4LVG4_ENTHI | Actophorin, putative EHI_197480 actin binding; intracellular | 29.71% | 24.82 | 15.7 |
| Q9BLF3_ENTHI | Rab family GTPase EhRab1A GTP binding; protein transport; small GTPase mediated signal transduction | 18.54% | 11.01 | 22.9 |
| B1N371_ENTHI | F-actin capping protein subunit beta, putative EHI_134490 F-actin capping protein complex; actin binding; actin cytoskeleton organization; cytoplasm | 18.24% | 7.05 | 19.4 |
| C4LX92_ENTHI | Rho family GTPase EHI_013260 GTP binding; intracellular; membrane; small GTPase mediated signal transduction | 17.09% | 18.65 | 22.3 |
| C4MB21_ENTHI | Profilin EHI_176140 actin binding; actin cytoskeleton; actin cytoskeleton organization; cytoplasm | 16.15% | 44.94 | 13.2 |
| C4LTB5_ENTHI | Actin-related protein 2/3 complex subunit 1A, putative EHI_045000 actin binding; cytoskeleton; regulation of actin filament polymerization | 15.30% | 14.09 | 40.5 |
| Q963D1_ENTHI | Rab family GTPase rab8 GTP binding; protein transport; small GTPase mediated signal transduction | 11.06% | 5.09 | 22.3 |
| C4LZW4_ENTHI | LIM zinc finger domain containing protein EHI_194520 zinc ion binding | 9.46% | 7.66 | 15.9 |
| C4LWU6_ENTHI | Calponin homology domain protein, putative EHI_199000 actin binding; calcium ion binding | 9.37% | 12.31 | 69.8 |
| C4LVQ1_ENTHI | Actin-binding protein, cofilin/tropomyosin family EHI_186840 actin binding; intracellular | 8.90% | 4.04 | 16.3 |
| C4M9S3_ENTHI | Rho family GTPase EHI_135450 GTP binding; intracellular; membrane; small GTPase mediated signal transduction | 8.04% | 2.86 | 22.4 |
| Q9NJW1_ENTHI | Grainin 2 grainin 2 calcium ion binding | 7.51% | 3.76 | 24 |
| C4M8K6_ENTHI | Rho family GTPase EHI_197840 GTP binding; intracellular; membrane; small GTPase mediated signal transduction | 6.67% | 7.34 | 21.6 |
| B1N4V4_ENTHI | Ras-like protein 3, putative EHI_049030 GTP binding; GTPase activity; intracellular; membrane; small GTPase mediated signal transduction | 5.88% | 2.51 | 19 |
| C4M855_ENTHI | Rho family GTPase EHI_052150 GTP binding; intracellular; membrane; small GTPase mediated signal transduction | 5.82% | 2.48 | 21.3 |
| C4MAF9_ENTHI | Grainin 1 EHI_167300 calcium ion binding | 5.12% | 3.78 | 24.3 |
| C4LT86_ENTHI | Importin alpha, putative EHI_044650 binding | 4.71% | 3.6 | 56.4 |
| B1N309_ENTHI | Rab GDP dissociation inhibitor alpha, putative EHI_167060 Rab GDP-dissociation inhibitor activity; protein transport | 2.96% | 2.77 | 49.3 |
| C4M782_ENTHI | ARP2/3 complex 34 kDa subunit, putative EHI_091250 cytoskeleton; regulation of actin filament polymerization | 2.73% | 2.49 | 33.6 |
| C4M943_ENTHI | Coronin, putative EHI_083590 | 2.31% | 2.59 | 48.1 |
